# Effect of static magnetic field on marine mollusc *Elysia leucolegnote*

**DOI:** 10.3389/fmolb.2022.1103648

**Published:** 2023-01-10

**Authors:** Fan Fei, Peng Zhang, Xinyu Li, Shun Wang, Erhui Feng, Yinglang Wan, Can Xie

**Affiliations:** ^1^ High Magnetic Field Laboratory, Hefei Institutes of Physical Science, Chinese Academy of Sciences, Hefei, Anhui, China; ^2^ University of Science and Technology of China, Hefei, Anhui, China; ^3^ Hainan Key Laboratory for Sustainable Utilization of Tropical Bioresources, College of Tropical Crops, Hainan University, Haikou, China; ^4^ Institutes of Physical Science and Information Technology, Anhui University, Hefei, Anhui, China; ^5^ Hainan Dong Zhai Gang National Nature Reserve Authority, Haikou, Hainan, China; ^6^ International Magnetobiology Frontier Research Center, Science Island, Hefei, China

**Keywords:** static magnetic field, geomagnetic field, oxidative stress, digestive, transcriptomics, apoptosis, *Elysia leucolegnote*

## Abstract

Artificial magnetic fields are unavoidable environment for offshore marine organisms. With the substantially increasing submarine cables, the impact of magnetic field generated by cables on marine organisms has gradually attracted people’s attention. However, there are few studies on the effect of magnetic field on molluscs. To explore whether magnetic fields could interfere with the physiological functions of offshore molluscs, here we systematically analyzed the change of metabolism and transcriptome of *Elysia leucolegnote* exposed to either geomagnetic field or 1.1 T static magnetic field. The blood glucose and lipid levels, as well as the activities of antioxidant enzymes in *E. leucolegnote* were significantly increased upon the exposure to high static magnetic field for 10 days. Meanwhile, the activities of enzymes related to digestive performance and liver functions were decreased. Possible mechanisms were further revealed through comparative transcriptome analysis. A total of 836 differentially expressed genes were identified, 352 of which were up-regulated and 484 of which were down-regulated after exposure to the high static magnetic field. The up-regulated differential genes were mainly concentrated in lysosomal and apoptotic pathways, and down-regulated differential genes were mainly involved in digestive and immune systems including phagocytosis. This pattern was further confirmed by RT-qPCR analysis. In conclusion, prolonged exposure to a 1.1 T static magnetic field increased oxidative stress and blood glucose and lipid levels, and decreased immunity and physiological conditions in *E. leucolegnote*. The data we presented here provides a comprehensive view of metabolism change and gene expression pattern of *E. leucolegnote* exposed to static magnetic field. It may expand our knowledge on the magnetic field effects on offshore mollusc at molecular level, and contribute to clarification of the interaction between marine animals and artificial magnetic fields, which is certainly ecologically important.

## Introduction

In the marine environment, both natural magnetic field and artificial magnetic fields coexist and permeate it. Earth’s magnetic field, also known as the geomagnetic field (GMF), is natural component of our environment, and is long-term and stable in nature, about 24–66 micro-Tesla (μT) (Alken et al., 2021). Many marine animals can detect the geomagnetic fields and utilize them in various important biological processes as orientation and navigation, which could be affected by artificially created magnetic fields (Cain et al., 2005). It has been showed previously that *Oncorhynchus nerka* can be deflected by 90° at night with an artificial magnetic field that rotates 90° in the horizontal component (Quinn, 1980); *Anguilla Anguilla* (Cresci et al., 2017), *Anguilla japonica* (Nishi and Kawamura, 2005) and *Thunnus albacares* (Nyqvist et al., 2020) can change the direction of migration by sensing the change of the magnetic field direction; Opisthobranch mollusc *Tritonia Diomedea* can derive directional cues from the geomagnetic field (Lohmann and Willows, 1987); *Piny lobsters* of the lobster family can determine their geographic location through the magnetic field parameters of the surrounding environment, and formulate a corresponding route to return to the habitat (Boles and Lohmann, 2003), which also appeared in the *Chelonia mydas* (Luschi et al., 2007); *Panulirus argus* can not only sense the direction of the magnetic field but also the inclination of the magnetic field (Ernst and Lohmann, 2016); *Zebrafish* showed bidirectional orientation in the geomagnetic field (Takebe et al., 2012).

In addition to influencing the behavior of marine organisms, magnetic field can also affect the physiology of marine organisms. Studies have shown that 600–2000 Gs can induce lipid peroxidation and reactive oxygen species (ROS) production in subcellular organelles and cells (Ishisaka et al., 2000). Increased mortality and significantly decreased biomass at 0.4–0.6 T magnetic field intensity were observed in *Silurus glanis* (Krzemieniewski et al., 2004). A strong static magnetic field of 14 T could affect the cleavage surface of *Xenopus eggs* (Denegre et al., 1998) and the positioning of mitogens (Valles, 2002) and a magnetic field of 4.05 T would significantly reduce the survival rate of *Neomysis awatschensis* (Yuan et al., 2016).

Due to the rapid scientific and technological progress, as well as the population growth, the number of offshore wind turbines, booster stations and submarine cables are also increasing day by day. Although the submarine cables located on the seabed has an insulating layer to shield the electric field, the electromagnetic disturbances cannot be eliminated completely. From land to shoal and then gradually deep into the ocean bottom sand, underwater cables can cause local deviations in the natural geomagnetic field (Taormina et al., 2018). For example, 1600 A electric currents could cause 320 μT at 1 m distance (Formicki and Winnicki, 1998), destroying the natural magnetic field in the vicinity, and affecting the life activities and living environment of coastal organisms and marine benthic organisms. Studies have shown that, migrating *European eels* in the Baltic Sea passing over an electric cable which inducing magnetic field strength of 5,000 nT at 60 m distance, deviated from their migration route (Nyqvist et al., 2020). The constant magnetic field of 1–13 mT caused the embryonic development of *Salmo trutta* and *Oncorhynchus mykiss* to be greatly slowed down (Formicki and Winnicki, 1998). The 10 mT and 1 mT magnetic field produced by the cable increased the *Oncorhynchus mykiss* yolk sac absorption rate, potentially negatively affecting the first feeding efficiency and subsequent growth rate (Fey et al., 2019). The electromagnetic field near the submarine cable at 50 Hz and 1 mT significantly reduced the ammonia excretion rate of the sand flea *Hediste diversicolor* but increased the burrowing activity (Jakubowska et al., 2019), and under the electrostatic magnetic field of 2.7 mT, it shows an escaping from the magnetic field of benthic (Bochert and Zettler, 2004).

Molluscs are the largest marine phylum, comprising about 23% of all the named marine organisms. Magnetic field can affect the behavior and physiology of molluscs too. Immune response was induced after exposure to an extremely low-frequency magnetic field (50 Hz, 100–500 μT) for 1 week in *Onchidium strumafor* (Zhang et al., 2020). The mitosis of the embryonic cells of *Paracentrotus lividus* could not carry out normal division or development exposed to the 6 mT magnetic field (Tenuzzo et al., 2016).

Among all the molluscs, *Elysia leucolegnote* is very special due to its photosynthetic ability (Maeda et al., 2021). This sea slug can integrate chloroplasts obtained during feeding into digestive cells through phagocytosis and perform photosynthesis to provide energy for their own life activities (Rumpho et al., 2009). Photosynthesis plays important roles in the life cycle of *E. leucolegnote* (Huang, 2004). The distribution of *E. leucolegnote* has been recorded in mangrove-dwelling bases in Philippines (Sanchez-Escalona, 2019), Singapore, Thailand, India (Swennen, 2011), Indonesia (Assuyuti and Wardiatno, 2020) and Hainan Province of China (Li et al., 2021). *E. leucolegnote* has soft body but without shell protection, and it is sensitive to changes of the external environment. Artificial magnetic fields are unavoidable environment for this offshore marine organism as well. How do magnetic fields affect the physiological conditions of *E. leucolegnote* remains unknown.

With an increase of anthropogenic pressure on ecosystems, the intensity of magnetic fields in the environment may increase in the future. Therefore, it is necessary to explore the effects of various magnetic field including high static magnetic field on marine organisms, such as molluscs. Meanwhile, higher magnetic field can amplify the relatively weak effects at both molecular and cellular level, thus reveal the underlying mechanism. In this study, we systematically analyzed the changes of metabolism and transcriptome of *E. leucolegnote* exposed to either geomagnetic field (GMF) or 1.1 T static magnetic field (SMF). After the exposure to SMF for 10 days, we found that the blood glucose and lipid levels, and the activities of antioxidant enzymes in *E. leucolegnote* were significantly increased. In contrast, the activities of enzymes related to digestive performance and liver functions were decreased. Our study revealed several biological effects of static magnetic field including the oxidative stress, as well as the downregulated digestive and immune systems, were observed in *E. leucolegnote*. The data not only provides useful biological references for the construction of submarine cables, but also expands our knowledge on the interaction between magnetic fields and biological species such as mollusc at both molecular and cellular levels.

## Materials and methods

### Experimental conditions and sample collection

All *E. leucolegnote* collected in Qidiao Village (110°38′26″ E, 19°56′31″ N, Haikou, Hainan, China) were cultured in laboratory (117°16′72″ E, 31°91′38″ N, Hefei, Anhui, China) for 7 days before experiments, to allow the sea slugs to acclimate to the experimental conditions. *E. leucolegnote* were placed on 1.1 T neodymium iron boron (NdFeB) N38 permanent magnets (length× width× height: 5.8 × 4.8 × 3.8 cm), namely 1.1 T static magnetic field (1.1 T SMF). To measure the distributions of the magnetic fields at different positions, a magnet analyzer (FE-2100RD, Forever Elegance, China) was used to scan the SMF distribution above the magnets. Pilot tests were carried out to determine the suitable SMF treatment time. The preliminary data suggested that the mortality rate of sea slugs in 1.1 T SMF group reached 70% at 14 days, and decreased to 40% at 13 days. And all sea slugs survived after 10 days with 1.1 T magnetic field treatment. Thus, 10 days of SMF treatment was chosen to explore the cause of death and the biological effects of magnetic field treatment for this study. To minimize the experimental variations, the control group (here named geomagnetic field treatment group, GMF group, with inclination of 47.6765°, declination of -5.5585° and total field intensity of 49,892.9 nT, which corresponding to 0.49 Gauss) was placed on aluminium block of the same size as 1.1 T SMF, and keep distant from the 1.1 T SMF group to eliminate the magnetic disturbance from artificial magnetic field. A total of 20 *E. leucolegnote* with an average length 4 mm were randomly assigned to two groups and placed in a plastic Petri dish (length× width× height: 4.3 × 1.1 × 1.1 cm) filled with self-configured seawater with a salinity of 25, pH 8.1 and sealed with a sealer. *E. leucolegnote* were placed in each group in an air-conditioned room maintained at 25°C, water temperature 18°C, and follow with the changes of 12 h light: 12 h dark for consecutive 10 days. After experiments, *E. leucolegnote* from each group were quickly frozen at −80°C ultra-low temperature refrigerator individually until subsequent enzyme activity and transcriptomics analysis.

### Analysis of enzyme activity

Glucose (Glu), Triglyceride (TG), Total cholesterol (TCH), enzyme activity of Catalase (CAT), Superoxide dismutase (SOD), Glutathione (GSH), Glutathione peroxidase (GSH-PX), Lysozyme (LZM), Aspartate aminotransferase (AST), Alanine transaminase (ALT), Amylase (AMS), Pepsin (PEP), Lipase (LPS) and Trypsin (TRY) were measured using kits purchased from Nanjing Jiancheng Bioengineering Institute (Nanjing, China).

### RNA sequencing

Total RNA was extracted from the *E. leucolegnote* using TRIzol® Reagent according the manufacturer’s instructions (Invitrogen) and genomic DNA was removed using DNase I (TaKara). The integrity and purity of the total RNA quality was determined by 2100 Bioanalyser (Agilent Technologies) and quantified using the ND-2000 (NanoDrop Technologies). Only high-quality RNA sample (OD 260/280 = 1.8–2.2, OD 260/230 ≥ 2.0, RIN≥ 6.5, 28 S: 18 S ≥ 1.0, >1 μg) was used to construct sequencing library.

Isolation of mRNA by oligo (dT) beads following the polyA selection method, and sequencing libraries were constructed by the TruSeqTM RNA sample of preparation Kit (Illumina, San Diego, CA). The first strand cDNA was synthesized using reverse transcriptase, random primers, and the short fragments, followed by second strand cDNA synthesis. Secondly double-stranded cDNA was synthesized using a SuperScript double-stranded cDNA synthesis kit (Invitrogen, CA) with random hexamer primers (Illumina). Then, the synthesized cDNA was subjected to end-repair, phosphorylation and “A” base addition. Libraries were size selected for cDNA target fragments of 300 bp on 2% Low Range Ultra Agarose followed by PCR amplified using Phusion DNA polymerase (NEB) for 15 PCR cycles. After quantified by TBS 380, paired-end RNA-seq sequencing library was sequenced with the Illumina NovaSeq 6000 sequencer (2 × 150 bp read length).

### Transcriptomic analysis

The clean reads data were obtained from the raw reads trimmed. All clean data from the samples were used to do de-novo assembly with Trinity (Grabherr et al., 2011). Then the assembled transcripts were assessed and optimized with BUSCO (Benchmarking Universal Single-Copy Orthologs) (Manni et al., 2021), TransRate (Smith-Unna et al., 2016) and CD-HIT (Fu et al., 2012). All the assembled transcripts were searched against the NCBI protein non-redundant, Swiss-Prot12, Pfam, Clusters of Orthologous Groups of proteins, GO (Gene Ontology) and KEGG (Kyoto Encyclopedia of Genes and Genomes) databases using BLASTX to identify the proteins that had the highest sequence similarity with the given transcripts to retrieve their function annotations and a typical cut-off E-values less than 1.0 × 10^−5^ was established.

To identify DEGs (differential expression genes) between two different treatments, the expression level of each gene was calculated according to the transcripts per million reads (TPM) method. RSEM (Li and Dewey, 2011) was used to quantify gene abundances. Essentially, differential expression analysis was performed using the DESeq2 (Love et al., 2014) with |log_2_ (foldchange)| ≥ 1 and P-adjust ≤.05. Functional-enrichment analysis including GO and KEGG were performed to identify which DEGs were significantly enriched in GO terms and metabolic pathways at P-adjust ≤.05 compared with the whole-transcriptome background. GO functional enrichment and KEGG pathway analysis were performed using Goatools and KOBAS (Xie et al., 2011), with adjusted *p* < .05 using the Benjamini–Hochberg method.

### Validation of differentially expressed genes by RT-qPCR

Eight DEGs from transcriptomic studies were randomly selected, and one gene (SSR3) with no expressional differences was chosen to validate the RNA-seq results by RT-qPCR. A housekeeping gene β-actin was used as an internal control to normalize the expression of the target genes. Primers for RT-qPCR were designed using Primer Premier 5.0 software, and primers’ details were listed in Supplementary Table S1. Total mRNA of the samples was extracted using above-mentioned method. Then, cDNA was synthesized using the PrimeScript^®^ RT Reagent Kit with gDNA Eraser (Takara, China), according to the manufacturer’s protocol. The SYBR Green RT-PCR assay was conducted to determine mRNA expressions of the genes. The temperature programming conditions were: denaturation at 95°C for 30 s, followed by 35 cycles of 95°C for 10 s, 60°C for 30 s, 95°C for 15 s, 60°C for 60 s, and 95°C for 1 s the melting curve was analyzed to confirm the presence of a single product in these reactions. Gene expression results were obtained using the 2^−ΔΔCT^ method (Livak and Schmittgen, 2001).

### Statistical analysis

There are at least three biological replicates, excluding RNA-seq, for each sample. GraphPad Prism five was used for histogram and statistical analysis. Student’s t-test was used to examine the raw data. Differences were considered significant at *p < .05.

## Results

### Blood glucose and lipid level of *E. leucolegnote* increased upon SMF treatment

**FIGURE 1:**
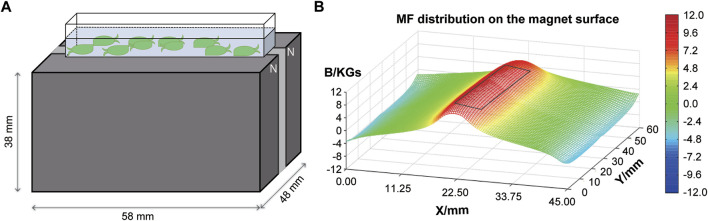
A schematic representation of the experimental setup. **(A)** Illustration of *E. leucolegnote* exposed to SMF provided by a permanent magnet. **(B)** Magnetic field distribution on the magnet surface measured by a magnet analyzer. The black rectangle represents the area where *E. leucolegnote* were placed.

**FIGURE 2:**
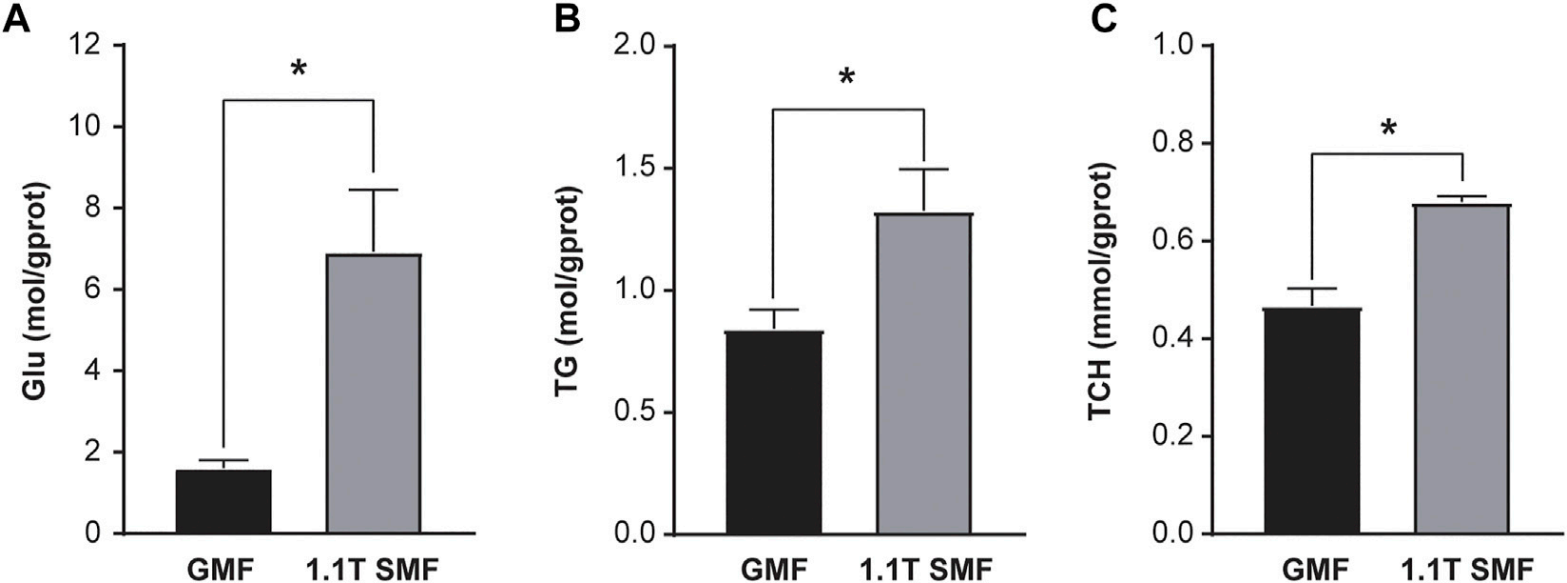
The effect of 1.1 T static magnetic field on blood glucose and lipid of *E. leucolegnote*. Glucose levels **(A)**, Triglyceride levels **(B)** and Total cholesterol levels **(C)** of *E. leucolegnote* were compared between the geomagnetic field group and 1.1 T static magnetic field group by Student’s t-test. *p < 0.05.

*E. leucolegnote* in SMF group were exposed to static magnetic field generated by permanent magnets as shown in Figure 1A. The surface of SMF distribution is uneven and ranges from 0.1 T to 1.1 T. The mollusc were placed in the middle area (black rectangle in Figure 1B), where the intensity of magnetic field could reach the highest 1.1 T. In this study, we firstly measured the glucose and lipid metabolism of *E. leucolegnote* and compared them in both GMF and SMF groups. Significant increase of blood glucose and lipid levels were observed after 10 days of SMF exposure (Figure 2, *p* < .05), represented by the increased glucose (Glu, Figure 2A), triglyceride (TG, Figure 2B) and total cholesterol (TCH, Figure 2C).

### SMF exposure increased the oxidative stress

**FIGURE 3:**
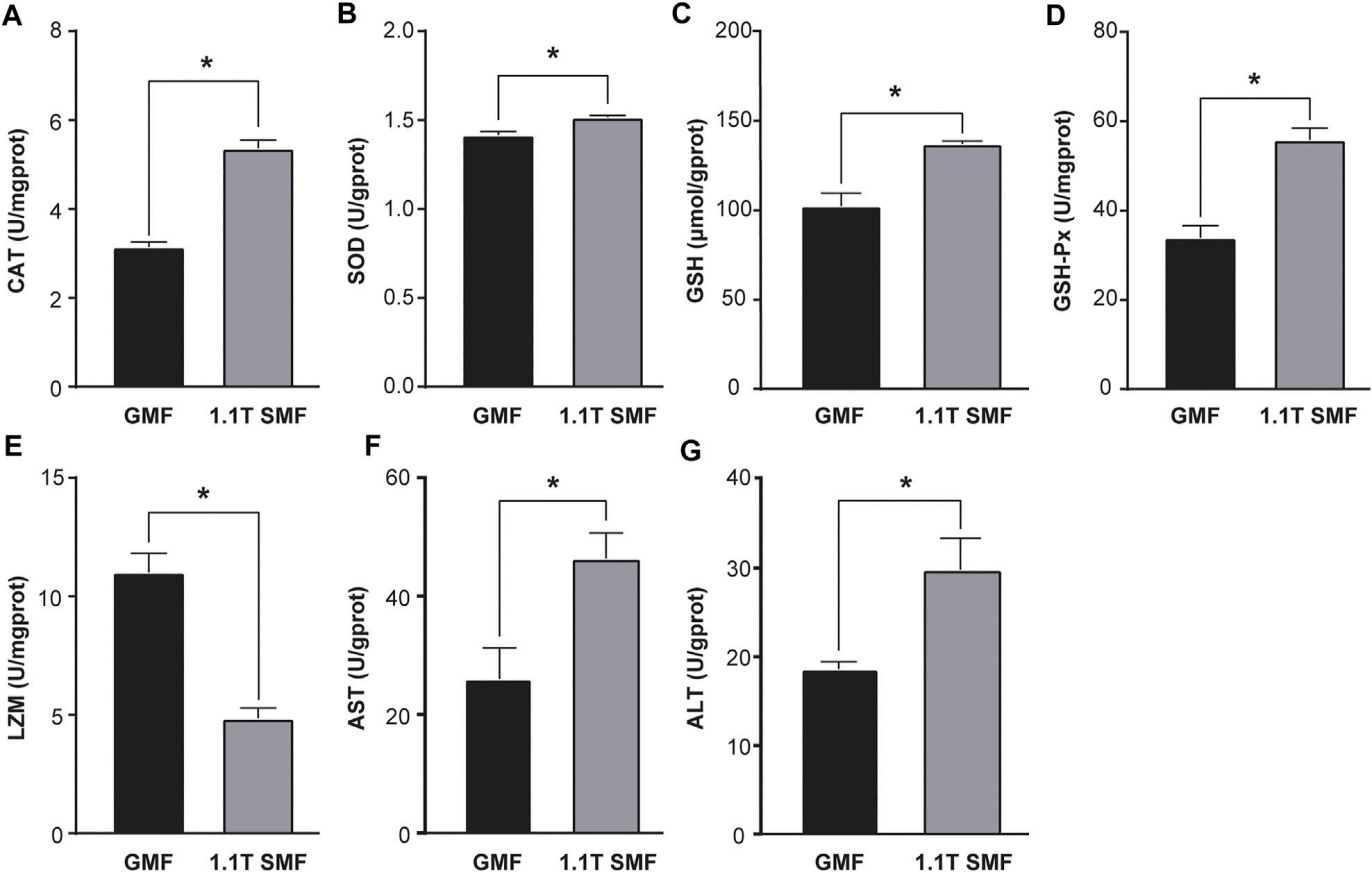
The 1.1 T static magnetic field caused oxidative stress in *E. leucolegnote*. Enzyme activities of catalase **(A)**, superoxide dismutase **(B)**, glutathione **(C)**, glutathione peroxidase **(D)**, lysozyme **(E)**, aspartate aminotransferase **(F)** and alanine transaminase **(G)** of *E. leucolegnote* exposed to 1.1 T SMF and GMF group were measured and compared. All comparisons were made between the GMF group and SMF group by Student’s t-test. *p < .05.

Static magnetic field treatment has significant effects on oxidative stress in *E. leucolegnote*. Obvious changes of antioxidant enzyme activities upon exposure to 1.1 T SMF were observed (Figure 3). The activity of several key antioxidant enzymes including CAT (Figure 3A), SOD (Figure 3B), GSH (Figure 3C) and GSH-PX (Figure 3D) in the 1.1 T SMF group were significantly higher than those in the GMF group (*p* < .05), while the LZM activity of 1.1 T SMF group was lower than those in GMF group (*p* < .05) (Figure 3E). And the activities of AST (Figure 3F) and ALT (Figure 3G) in the 1.1 T SMF group were significantly higher than those in GMF group (*p* < .05).

### Decreased digestive performance upon SMF exposure

**FIGURE 4:**
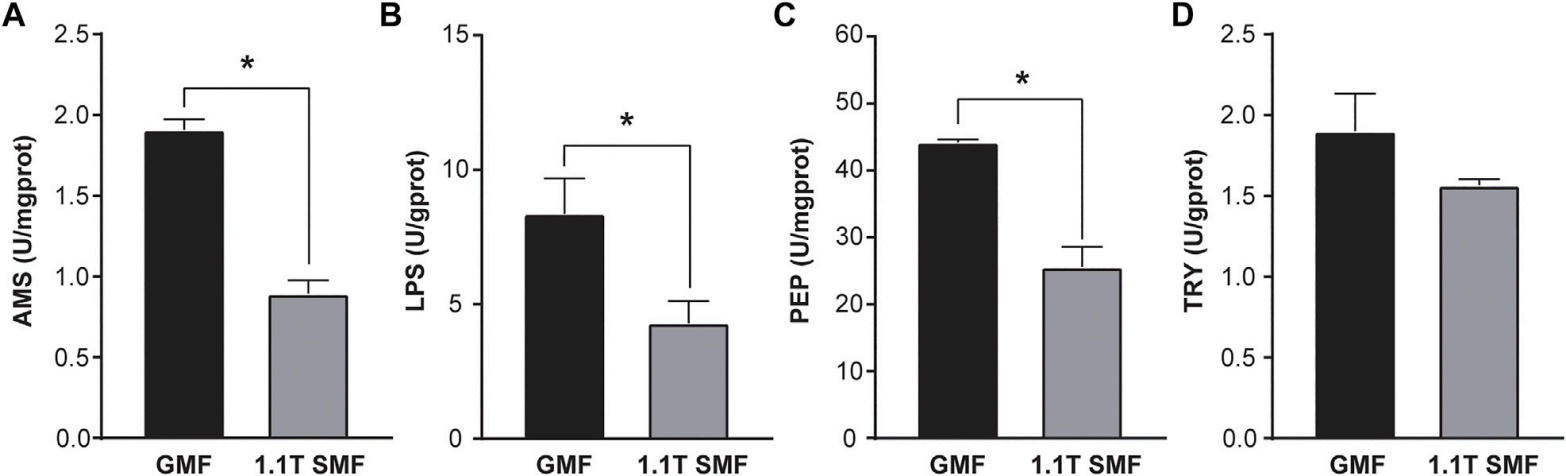
The effect of 1.1 T static magnetic field on digestive enzyme activity of *E. leucolegnote*. The enzyme activities of amylase **(A)**, lipase **(B)** pepsin **(C)** and trypsin **(D)** of *E. leucolegnote* exposed to 1.1 T SMF and GMF were measured and compared. All comparisons were made between the GMF group and SMF group by Student’s t-test. *p < .05.

Magnetic field has a significant impact on digestive enzyme activity as well (Figure 4). Several enzymes related to digestive performance of digestive glands including AMS (Figure 4A), PEP (Figure 4B) and LPS (Figure 4C) were down-regulated in the 1.1 T SMF group, compared with those in GMF group (*p* < .05), but no significant differences in TRY activity were detected among two groups (*p* > .05, Figure 4D). Since AMS, PEP and LPS are associated with the primary macronutrients in our diet, such as carbohydrates, proteins, and fats respectively, the activity of these enzymes represents a foundational aspect of gastrointestinal health. The decreased activity of these enzymes suggested 1.1 T SMF exposure led to decreased digestive performance in *E. leucolegnote*. The same finding was seen for the downregulated genes in the transcriptome as shown below.

### Comparative transcriptomic analysis

To further investigate the possible molecular mechanism of the magnetic effects on *E. leucolegnote*, a comparative analysis of the gene expression profiles was performed. We sequenced and compared the transcriptome of *E. leucolegnote* exposed to either the 1.1 T static magnetic field or the geomagnetic field. More than 300 million raw were obtained, and an average of 49.7 and 47.6 million clean reads were localized to the *E. leucolegnote* genome from the different magnetic field treatment, respectively (Table 1). The GC contents of two groups showed the values with 43.96% and 43.36%. And Q20 (those with a base quality greater than 20) contents were in the average of 97.78% and 97.68%, Q30 (those with a base quality greater than 30) contents were in the average of 93.63% and 93.40% (Table 1). These data suggested that the quality of sequencing results was sufficiently high and reliable for the subsequent transcriptome analysis.

**TABLE 1:** Statistics of E. leucolegnote transcriptome sequences.

Sample	Raw reads	Raw bases	Clean reads	Clean bases	Error rate (%)	Q20 (%)	Q30 (%)	GC content (%)
GMF_3	53171544	8.03E+09	51985634	7.64E+09	0.0258	97.7	93.43	43.43
GMF _2	43546366	6.58E+09	42263228	6.22E+09	0.0254	97.85	93.8	44.71
GMF _1	56586180	8.54E+09	54894802	8.01E+09	0.0256	97.8	93.67	43.73
1.1 T MF_3	50169004	7.58E+09	49030466	7.17E+09	0.0255	97.83	93.72	42.79
1.1 T MF_2	51743906	7.81E+09	50300764	7.38E+09	0.0261	97.58	93.18	43.42
1.1 T MF_1	44863688	6.77E+09	43489206	6.36E+09	0.026	97.62	93.31	43.87

**FIGURE 5:**
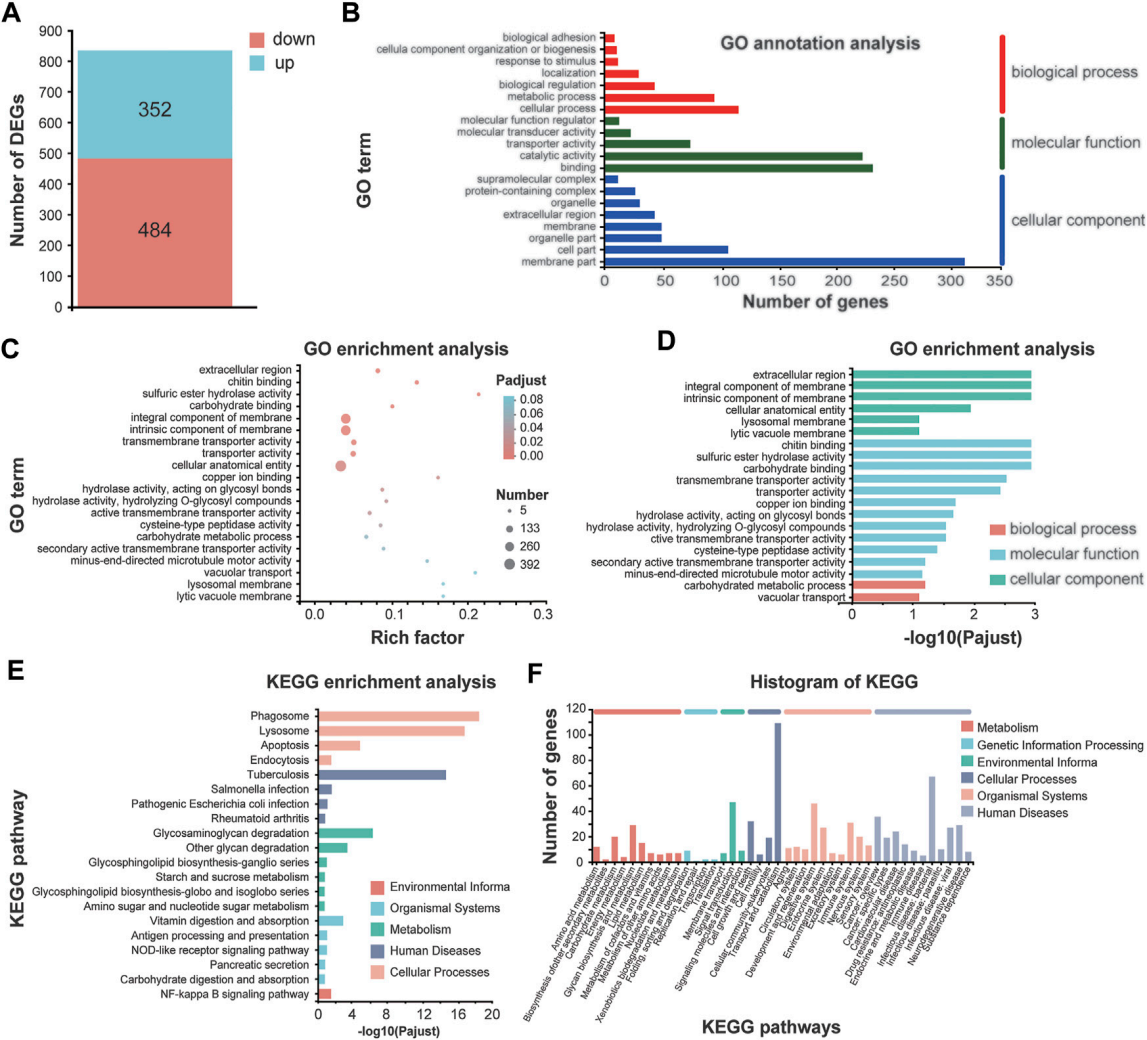
Comparative transcriptomic analysis of *E. leucolegnote* exposed to 1.1 T static magnetic field and geomagnetic field. **(A)** The number of up- and down-regulated DEGs were identified in the 1.1 T SMF and GMF treatment. **(B–F)** GO term annotation analysis **(B)**, GO term enrichment analysis **(C, D)** and KEGG enrichment analysis **(E, F)** to mapping the DEGs in response to 1.1 T SMF treatment compared with GMF treatment. Pathway enrichment analysis plots (top 20) of expressed metabolisms according to *p* < .05.

In total, 836 differentially expressed genes (DEGs) were identified in 1.1 T SMF treatment compared with GMF group, among which 352 genes are upregulated, and 484 genes are downregulated (Figure 5A and Supplementary Table S2). To identify the processes enriched in significant DEGs, we subjected significant DEGs to gene ontology (GO) functional annotation and term enrichment analysis, a tool developed to represent common and basic biological information. Most DEGs were involved in the biological processes such as cellular process and metabolic processes, catalytic activity and binding in the molecular function, and cell part and membrane part in the cellular component. In our study, GO enrichment analysis corresponding to 836 significant DEGs were produced and assigned to 69 functional groups and three categories (Figure 5B). Most of DEGs in the comparison of 1.1 T SMF vs. GMF were enriched in cellular component such as “extracellular region,” “integral component of membrane,” “intrinsic component of membrane” and “cellular anatomical entity,” and three additional in molecular function such as “chitin binding,” “sulfuric ester hydrolase activity” and “carbohydrate binding” (Figures 5B–D). Mapping these DEGs to the pathways from databases KEGG suggested that these genes are significantly clustered into several key signaling pathways, namely phagosome lysosome, apoptosis and endocytosis in cellular processes, *tuberculosis* in human diseases, glycosaminoglycan degradation and other glycan degradation in metabolism, vitamin digestion and absorption in organismal systems and NF-kappa B signaling pathway in environmental information (Figures 5E, F).

**FIGURE 6:**
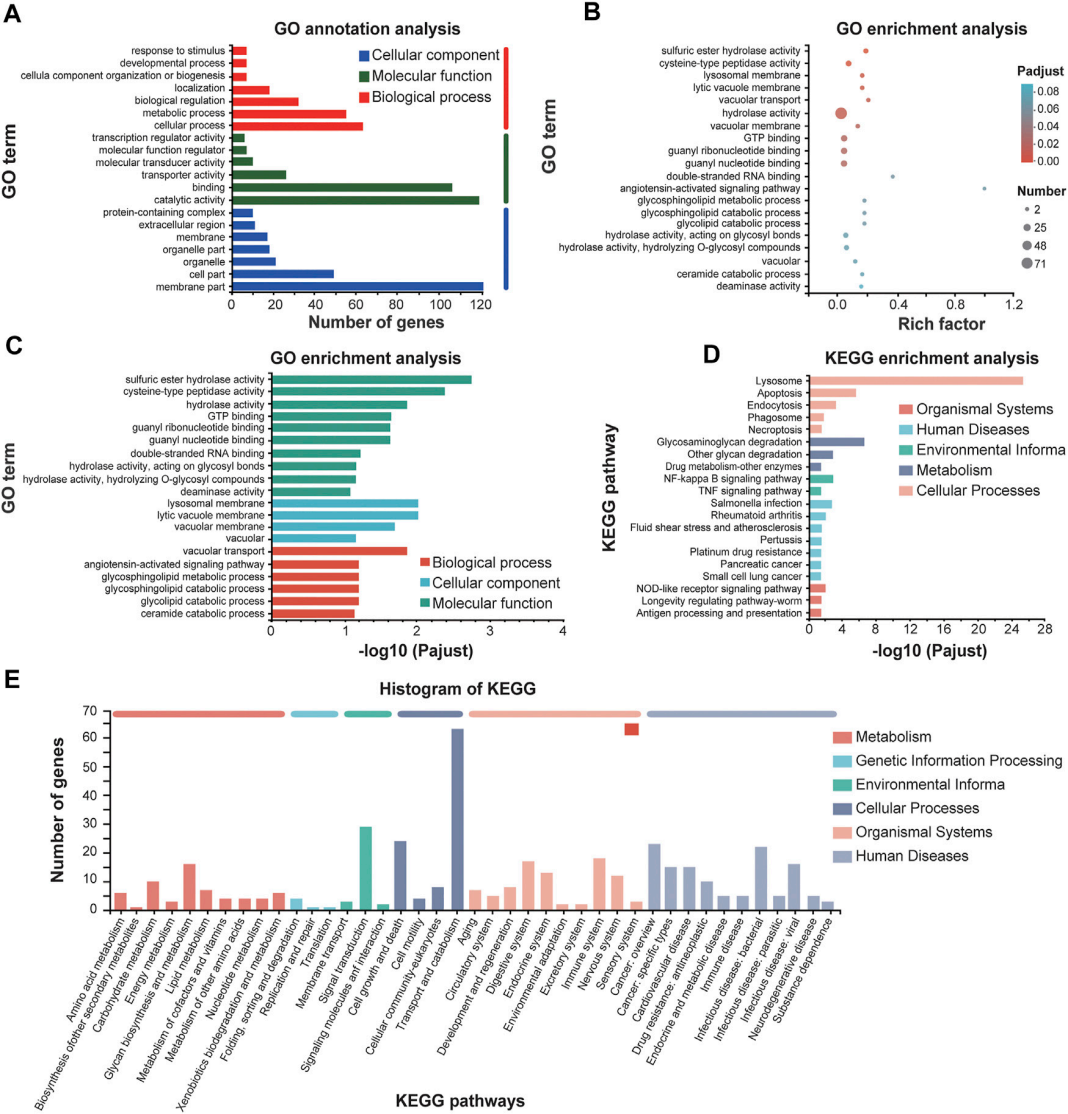
GO annotations and KEGG enrichment analysis of upregulated genes of *E. leucolegnote* under 1.1 T static magnetic field. GO term annotation analysis **(A)**, GO term enrichment analysis **(B, C)** and KEGG enrichment analysis **(D, E)** of upregulated genes in response to 1.1 T SMF and GMF treatment were analyzed and compared. Pathway enrichment analysis plots (top 20) of expressed metabolisms according to *p* < .05.

Significantly upregulated genes and downregulated genes were selected based on the annotation of DEGs. The upregulated genes were significantly clustered into biological processes including “vacuolar transport,” “sulfuric ester hydrolase activity,” “cysteine-type peptidase activity” and “hydrolase activity” in molecular function, and “lysosomal membrane” and “lytic vacuole membrane” in cellular component (Figures 6A–C). Mapping these upregulated genes to the pathways from databases KEGG suggested that these upregulated genes are significantly enriched in the signaling pathways of lysosome and apoptosis in cellular processes (Figures 6D, E). It is worth pointing out that both the caspase and lysosome change of *E. leucolegnote* after 10 day of SMF treated are related and involved in apoptosis pathway based on the apoptosis map, this further validates the involvement of lysosomal proteases (Turk et al., 2002) and caspase family (Bearoff and Fuller-Espie, 2011) in the apoptotic pathways.

Taking together, our data suggested that the upregulated genes upon SMF treatment identified in this study were mostly leading to apoptosis and increased lysosomal activity, which is consistent with our biochemical studies as shown in Figures 3, 4, and in an agreement with previous reports as well. For example, SMF exposure alone or in combination with extremely low frequency MF (ELF) of more than 1 mT have a selective impact on cell signaling related to apoptosis, which may through magnetic field effect on motion of charged molecules (Tofani et al., 2001). In another study, after 12 days of exposure to the 1 mT electromagnetic field generated by the submarine cable, the number of apoptotic in the *Limecola balthica* was significantly elevated (Stankeviciute et al., 2019). It has been suggested that the underlying mechanism of how magnetic field affect apoptosis may through magnetic field effect on electron spin, which lead to increased reactive oxygen species (ROS) concentration (Tofani, 2022).

**FIGURE 7:**
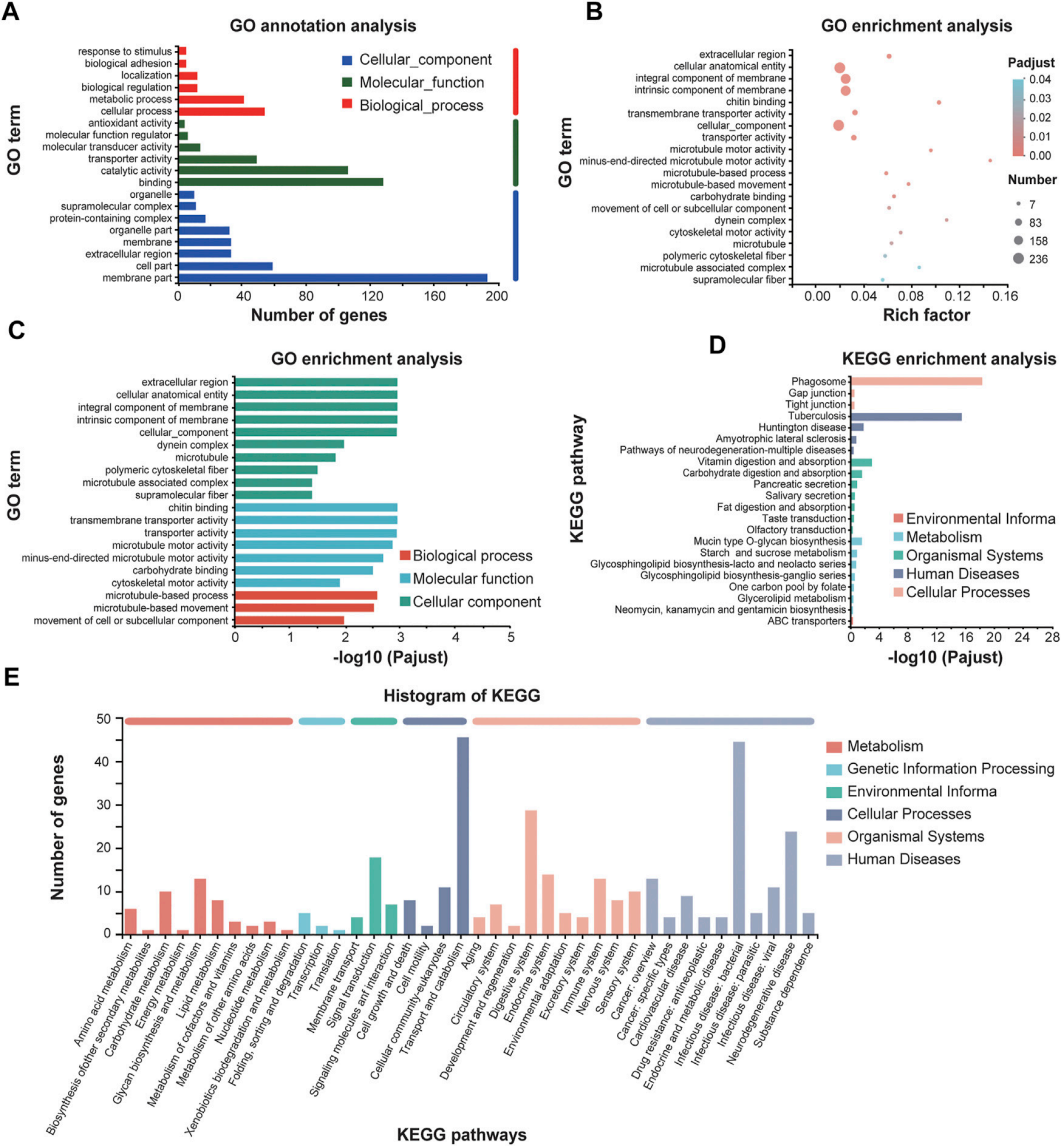
GO annotations and KEGG enrichment analysis of downregulated genes of *E. leucolegnote* under 1.1 T static magnetic field. GO term annotation analysis **(A)**, GO term enrichment analysis **(B, C)** and KEGG enrichment analysis **(D, E)** of downregulated genes in response to 1.1 T SMF and GMF treatment were analyzed and compared. Pathway enrichment analysis plots (top 20) of expressed metabolisms according to *p* < .05.

We also analyzed the downregulated genes by GO annotations and KEGG enrichment analysis (Figure 7). GO functional enrichment showed that most downregulated genes were enriched in molecular function such as “chitin binding” and “transmembrane transporter activity,” and in cellular component such as “extracellular region,” “cellular anatomical entity,” and “microtubule-based process” and “microtubule-based movement” as well in biological process (Figures 7A–C). Mapping these DEGs to the pathways from databases KEGG suggested that these downregulated genes are significantly clustered into the signaling pathways of phagosome, digestive systems and infectious diseases (Figures 7D, E).

Taking together, our data suggested that among the total of 484 downregulated genes, many of them might lead to the reduced digestive performance and immune systems, which could increase the risk of infectious diseases. The analysis of downregulated DEGs is largely consistent with our metabolism studies as shown in Figures 3, 4.

### Validating the accuracy of the transcriptome expression results using quantitative real time PCR

**FIGURE 8:**
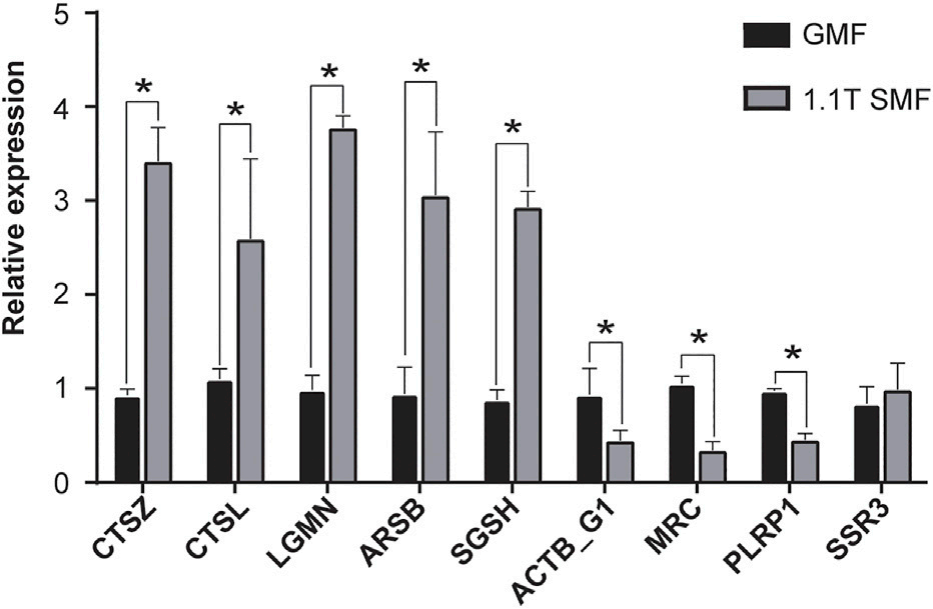
Validation of the transcriptome expression pattern RT-qPCR. The expression levels of selected genes (see details in text) were measured and compared between 1.1 T SMF group and GMF group. Each normalized to that of the geomagnetic field group. All comparisons were made by Student’s t-test. *p < .05.

To further validate the accuracy and reliability of transcriptomic data, eight DEGs from transcriptomic studies including significantly up/down regulated genes, were chosen, and one gene with no expressional differences were used as control. Real-time quantitative PCR (RT-qPCR) was carried out on these nine genes to measure the expression levels (Figure 8). Notably, five DEGs involved in apoptosis and lysosome pathways such as cathepsin Z (CTSZ), cathepsin-L (CTSL), vacuolar-processing enzyme (LGMN), arylsulfatase B (ARSB) and N-sulphoglucosamine sulphohydrolase (SGSH) showed significant up-regulation. In contrast, two DEGs involved in phagosome pathway such as actin G1 (ACTB_G1) and macrophage mannose receptor 1 (MRC) and one DEG involved in Glycerolipid metabolism pathway, pancreatic lipase-related protein (PLRP1), appeared to be down-regulated. And one DEG involved in Protein processing in endoplasmic reticulum pathway, translocon-associated protein subunit gamma (SSR3), only showed mild change in RT-qPCR validation. The data suggested the several signaling pathways including apoptosis, lysosome and phagosome pathway were clearly involved in the SMF response, which shed light on the poorly understand molecular mechanism of magnetic field effects on biological organisms.

## Discussion

Although knowledge of how animals use geomagnetic field is increasing, benefited from the rapid and significant progress over the past decades in animal magnetoreception (Johnsen and Lohmann, 2008; Qin et al., 2016; Mouritsen, 2018; Xie, 2022), essential knowledge regarding to how marine animals interact with artificial electromagnetic fields is still missing. Extensive field studies have been applied to demonstrate how magnetic field can affect the behavior, development, and physiology of marine animals, or change the migration route of migrating species. In tandem with field studies, well controlled laboratory experiments should also be carried out to investigate molecular mechanism underlying the biological effects of magnetic fields on marine animals.

Biochemical studies were applied to evaluate the effects of SMF on the metabolism of cell cultures, animals, and humans previously, but not in marine mollusc. We start by analyzing the change of metabolism of *E. leucolegnote* exposed to 1.1 T SMF compared with the sea slug under geomagnetic field. As a primary energy molecule, glucose is extremely sensitive to different levels of stress and is regulated according to feedback mechanisms (Jiang et al., 2017). Previous studies showed that, stress was positively correlated with blood glucose (Wells and Pankhurst, 1999) and the upregulation of TG content and promoted lipid storage (Ling et al., 2020; Yu et al., 2021). In our study, the blood glucose and lipid level were significantly increased in *E. leucolegnote* exposed to 1.1 T SMF, which is in agreement with previous reports. It is possible that the sea slug was increasing the content of blood glucose and blood lipids to provide energy to cope with the external environmental pressure, such as artificial magnetic fields.

The dynamic equilibrium between the antioxidant system and ROS are essential for biological organisms (Fei et al., 2020a). Antioxidant enzymes such as SOD, CAT, GSH and GSH-Px play key roles in protecting cells from oxidative stress by countering the toxic effects of reactive oxygen species (ROS) (Fei et al., 2020b). It has been reported that magnetic field increased ROS levels in human, mouse, rat cells, and tissues (Ishisaka et al., 2000; Wang and Zhang, 2017; Yu et al., 2021). Consistently, the activities of antioxidant enzymes in *E. leucolegnote* were significantly increased upon the exposure to 1.1 T high magnetic field for 10 days in this study, indicating that changes in the magnetic field environment may trigger the production of a large amount of ROS, which result in a certain oxidative stress.

We also found that 1.1 T SMF caused possible liver damage, decreased antibacterial and immune function in *E. leucolegnote*, represented by the increase of enzyme activity of ALT, AST and decrease of LZM. Similar phenomena have been reported in many aquatic organisms previously. For example, studies showed that, the lysozyme level significantly decreased, and the AST and ALT levels significantly increased upon magnetic field exposure in *Cyprinus Carpio* (Khoshroo et al., 2018) and *Caspian kutum* (Loghmannia et al., 2015).

Magnetic field exposure also leads to impaired digestive performance in *E. leucolegnote*, as shown by the reduced activity of AMS, PEP and LPS. The digestive system is particularly important for the viability of organisms. Different magnetic field exposure may have different effects on different organisms, based on previous studies. For example, hypomagnetic conditions could reduce the activity of digestive enzymes in *Carassius Carassius* (Kuz’mina et al., 2015) and in *Rutilus rutilus* (Golovanova et al., 2021), but when the magnetic field increase to 150 μT magnetic field, the digestive enzyme activity in *Rutilus rutilus* increased (Golovanova et al., 2013); Pepsin activity in *Wistar rats* was stimulated by a D-Polarization rotated electromagnetic field generated by a 37 GHz electromagnetic field, whereas pepsin production was suppressed by a L-Polarization magnetic field (Subbotina et al., 2004). Here in this study, we used 1.1 T SMF to treat the sea slug *E. leucolegnote*, and significantly reduced digestive enzyme activities was observed. It is worth pointing out that two groups of *E. leucolegnote* did not take any food during the experiment due to the lacking information of food source of *E. leucolegnote*, thus the photosynthesis might be the only way to provide energy. Therefore, it is possible that reduction of activity of digestive enzymes would help *E. leucolegnote* to maintain essential life activities and cope with the external environmental pressure caused by magnetic field.

The comparative transcriptome study of *E. leucolegnote* exposed to SMF and GMF further confirmed the data we derived from biochemical and metabolism studies. The increased activity of antioxidant enzymes servers as an indicator of ROS production and inflammation, directly or indirectly damage cells, and eventually induce cell death (Simpson and Oliver, 2020), and the decreased liver function and digestive function are partly attributed to apoptosis caused by oxidative stress or cell damage (Emre et al., 2011). Consistently, in this study, our subsequent transcriptome analysis revealed that the enrichment of upregulated gene significantly clustered into apoptosis pathway and increased lysosomal activity, whereas the downregulated genes were enriched to the digestive and immune systems.

In all, the 1.1 T static magnetic field treatment had a negative impact on *E. leucolegnote*. Similar negative effects of magnetic field have been observed in many aquatic organisms such as *Limecola balthica* (Stankeviciute et al., 2019), *Silurus glanis* (Ernst and Lohmann, 2016), *Neomysisa watschensis* (Luschi et al., 2007), *Sparus macrocephalus* (Luschi et al., 2007), *Cancer pagurus* (Scott et al., 2021) and *Limecola balthica* (Stankeviciute et al., 2019). Based on our study, the significant increase of lysosomes enrichment and activation of apoptosis pathway were observed when *E. leucolegnote* exposed to 1.1 T static magnetic field, which could represent important strategies for animal survival under stress by eliminating the redundant or damaged organelles. The purpose here, therefore, is to provide an overview of biological effects of magnetic fields on offshore molluscs such as *E. leucolegnote* at molecular level, which would provide us clues to evaluate how artificial magnetic fields may interact with and affect marine animals.

## Data Availability

The datasets presented in this study can be found in online repositories. The names of the repository/repositories and accession number(s) can be found below: http://www.ncbi.nlm.nih.gov/bioproject/904688.
