# Supplementary material for: Effect of static magnetic field on marine mollusc Elysia leucolegnote
Source: Front Mol Biosci. 2023 Jan 10;9:1103648. doi: 10.3389/fmolb.2022.1103648 (PMC9871387; doi:10.3389/fmolb.2022.1103648)
Supplement: Supplementary file 1 [file DataSheet1.PDF]

Supplementary Material

Supplementary Tables

Supplementary Table 1. Primer sequences for the genes used for RT-qPCR.

Gene name	Primer sequences (5'-3')	
	Forward primers	Reverse primers
ACTB_G1	CCCAAGATTTGCTCATAGA	TCAGCCATAACACGGACAC
CTSZ	CGCCATCTATCAACCACA	GGAACCACCCAACCTCAC
ARSB	TGGCGGCACCATTTACTA	GGTTTCGTCCCGTTCAGG
CTSL	CCTCTGGAGCCACATACA	GCAGTTGCGGTTGGGTTG
SGSH	TGGGTGTTATTCCTGACT	ATTCTCCCTATGGTTGTG
LGMN	TGAGGCAGAGGAAGAGTG	AAGGCATAGTAGTTCTGTGGTA
MRC	GAAACAAGCCTGTAACCC	CAACTGTCACTAAACCGAGA
PLRP1	TCATACATACAGACGACGAGC	GCATTTCCGAGTGTAGAGTAGA
SSR3	ATTGGCAGATGATAAGCG	CAGGTACAGGGCATTGTT
β-actin	ATCCACGAGACCACCTAC	AGCCTCCAATCCATACTG

Supplementary Table 2. Summary statistics of *E. leucolegnote* transcriptome and differentially expressed genes based on blast assignment.

Gene_id	Gene_name	1.1T SMF vs GMF	Sum
OV30007635.g	maker-chr3-augustus-gene-59.7	yes|up	1
OV80031790.g	maker-chr8-snap-gene-49.13	yes|up	1
OV10026250.g	maker-chr1-snap-gene-1037.50	yes|down	1
OV110031104.g	maker-chr11-snap-gene-247.31	yes|down	1
OV70021760.g	maker-chr7-snap-gene-349.1	yes|down	1
OV110031541.g	maker-chr11-snap-gene-318.41	yes|up	1
OV20016599.g	maker-chr2-augustus-gene-1070.29	yes|up	1
OV100028265.g	maker-chr10-augustus-gene-191.21	yes|up	1
OV40002835.g	maker-chr4-snap-gene-313.8	yes|up	1
OV30007975.g	maker-chr3-snap-gene-136.24	yes|up	1
OV100028713.g	maker-chr10-snap-gene-312.0	yes|down	1
OV140017245.g	maker-chr14-augustus-gene-131.16	yes|down	1
OV10027187.g	maker-chr1-snap-gene-1281.19	yes|down	1
OV30008858.g	maker-chr3-augustus-gene-355.16	yes|up	1
OV80032993.g	maker-chr8-augustus-gene-291.5	yes|down	1
OV150028893.g	maker-chr15-exonerate_est2genome-gene-18.1	yes|up	1
OV70021896.g	maker-chr7-snap-gene-379.12	yes|down	1
OV10023456.g	maker-chr1-snap-gene-321.4	yes|up	1
OV30010093.g	maker-chr3-snap-gene-646.8	yes|down	1
OV10024839.g	maker-chr1-snap-gene-692.47	yes|down	1

OV10024060.g	maker-chr1-augustus-gene-486.22	yes|up	1
OV40003254.g	maker-chr4-snap-gene-415.16	yes|down	1
OV10023283.g	maker-chr1-augustus-gene-277.2	yes|up	1
OV140017611.g	maker-chr14-snap-gene-215.32	yes|down	1
OV10024789.g	maker-chr1-augustus-gene-680.30	yes|down	1
OV120011073.g	maker-chr12-snap-gene-65.38	yes|up	1
OV140017181.g	maker-chr14-snap-gene-112.5	yes|down	1
OV110030841.g	maker-chr11-snap-gene-192.33	yes|down	1
OV130007172.g	maker-chr13-augustus-gene-263.20	yes|down	1
OV10023224.g	maker-chr1-exonerate_protein2genome-gene-266.18	yes|up	1
OV130006764.g	maker-chr13-snap-gene-169.3	yes|up	1
OV110031042.g	maker-chr11-snap-gene-236.1	yes|down	1
OV20014583.g	maker-chr2-snap-gene-578.22	yes|down	1
OV10025560.g	maker-chr1-snap-gene-871.10	yes|down	1
OV130007170.g	maker-chr13-snap-gene-262.0	yes|down	1
OV10026177.g	maker-chr1-snap-gene-1016.8	yes|up	1
OV50018790.g	maker-chr5-snap-gene-192.0	yes|up	1
OV10023478.g	maker-chr1-augustus-gene-326.66	yes|down	1
OV20016146.g	maker-chr2-snap-gene-952.23	yes|down	1
OV60004781.g	maker-chr6-augustus-gene-126.4	yes|up	1
OV100027817.g	maker-chr10-exonerate_protein2genome-gene-77.3	yes|up	1
OV10023105.g	maker-chr1-snap-gene-238.1	yes|down	1
OV70021053.g	maker-chr7-augustus-gene-215.3	yes|down	1

OV140017185.g	maker-chr14-augustus-gene-113.27	yes|down	1
OV140017108.g	maker-chr14-augustus-gene-90.67	yes|down	1
OV140017162.g	maker-chr14-augustus-gene-107.10	yes|up	1
OV20015166.g	maker-chr2-augustus-gene-718.1	yes|down	1
OV60005599.g	maker-chr6-exonerate_protein2genome-gene-311.7	yes|down	1
OV10024184.g	maker-chr1-snap-gene-517.2	yes|up	1
OV30007625.g	augustus-chr3-processed-gene-57.19	yes|up	1
OV40003272.g	maker-chr4-snap-gene-419.4	yes|down	1
OV20015842.g	maker-chr2-snap-gene-881.1	yes|down	1
OV50019148.g	maker-chr5-augustus-gene-277.43	yes|down	1
OV110031478.g	maker-chr11-snap-gene-305.45	yes|up	1
OV140016704.g	maker-chr14-snap-gene-8.41	yes|down	1
OV20014124.g	maker-chr2-exonerate_protein2genome-gene-468.29	yes|down	1
OV20013779.g	maker-chr2-snap-gene-377.19	yes|up	1
OV120011017.g	maker-chr12-snap-gene-47.55	yes|down	1
OV100028598.g	maker-chr10-snap-gene-284.41	yes|down	1
OV10026718.g	maker-chr1-snap-gene-1162.0	yes|up	1
OV20013263.g	maker-chr2-augustus-gene-248.19	yes|down	1
OV50019705.g	maker-chr5-snap-gene-392.2	yes|up	1
OV70020337.g	maker-chr7-augustus-gene-55.73	yes|down	1
OV30008121.g	maker-chr3-exonerate_est2genome-gene-169.27	yes|down	1
OV140017778.g	maker-chr14-snap-gene-247.11	yes|up	1

OV140016916.g	maker-chr14-snap-gene-44.42	yes|down	1
OV80031663.g	maker-chr8-augustus-gene-22.65	yes|up	1
OV20013336.g	maker-chr2-snap-gene-265.25	yes|down	1
OV10026017.g	maker-chr1-augustus-gene-983.3	yes|up	1
OV140017239.g	maker-chr14-augustus-gene-129.3	yes|up	1
OV50019099.g	maker-chr5-snap-gene-260.4	yes|down	1
OV60004816.g	maker-chr6-augustus-gene-133.38	yes|up	1
OV10027171.g	maker-chr1-snap-gene-1276.2	yes|down	1
OV130006959.g	maker-chr13-augustus-gene-208.11	yes|up	1
OV30009851.g	maker-chr3-augustus-gene-589.62	yes|down	1
OV30007619.g	maker-chr3-snap-gene-56.21	yes|down	1
OV60004271.g	maker-chr6-snap-gene-4.22	yes|down	1
OV130007097.g	maker-chr13-augustus-gene-245.54	yes|down	1
OV100028076.g	maker-chr10-snap-gene-144.15	yes|down	1
OV130006910.g	maker-chr13-snap-gene-201.67	yes|up	1
OV100027564.g	maker-chr10-snap-gene-17.52	yes|down	1
OV100028304.g	maker-chr10-snap-gene-202.33	yes|down	1
OV80032863.g	maker-chr8-augustus-gene-271.24	yes|down	1
OV140017206.g	maker-chr14-augustus-gene-122.37	yes|up	1
OV10023458.g	maker-chr1-snap-gene-322.48	yes|up	1
OV60005088.g	maker-chr6-snap-gene-199.4	yes|down	1
OV40003932.g	maker-chr4-augustus-gene-569.9	yes|up	1
OV40001892.g	maker-chr4-augustus-gene-67.0	yes|up	1

OV130007333.g	maker-chr13-snap-gene-305.35	yes|up	1
OV30010075.g	maker-chr3-augustus-gene-643.1	yes|down	1
OV140016789.g	maker-chr14-snap-gene-23.22	yes|down	1
OV110030292.g	maker-chr11-snap-gene-68.7	yes|up	1
OV80031645.g	maker-chr8-snap-gene-19.4	yes|up	1
OV40003373.g	maker-chr4-exonerate_est2genome-gene-441.3	yes|up	1
OV10024141.g	maker-chr1-augustus-gene-504.0	yes|down	1
OV20013668.g	maker-chr2-exonerate_est2genome-gene-351.5	yes|down	1
OV100027613.g	maker-chr10-augustus-gene-30.100	yes|down	1
OV50019122.g	maker-chr5-augustus-gene-267.7	yes|down	1
OV20013670.g	maker-chr2-snap-gene-351.8	yes|up	1
OV130006500.g	maker-chr13-snap-gene-106.13	yes|down	1
OV60005084.g	maker-chr6-snap-gene-198.14	yes|up	1
OV60005018.g	maker-chr6-snap-gene-181.4	yes|up	1
OV10022560.g	maker-chr1-snap-gene-113.36	yes|down	1
OV20015528.g	maker-chr2-snap-gene-805.33	yes|up	1
OV150028964.g	maker-chr15-snap-gene-29.24	yes|down	1
OV60004757.g	snap-chr6-processed-gene-117.13	yes|down	1
OV40003375.g	maker-chr4-exonerate_est2genome-gene-441.4	yes|up	1
OV140016742.g	maker-chr14-snap-gene-14.13	yes|up	1
OV10024744.g	maker-chr1-augustus-gene-664.1	yes|up	1
OV120010935.g	maker-chr12-snap-gene-27.2	yes|up	1
OV80032798.g	maker-chr8-augustus-gene-260.5	yes|down	1

OV40002833.g	maker-chr4-snap-gene-312.4	yes|down	1
OV10025558.g	maker-chr1-snap-gene-871.11	yes|down	1
OV130007007.g	maker-chr13-snap-gene-220.8	yes|down	1
OV30009747.g	maker-chr3-snap-gene-566.60	yes|up	1
OV80033102.g	snap-chr8-processed-gene-320.13	yes|down	1
OV90000305.g	maker-chr9-snap-gene-48.2	yes|down	1
OV150029576.g	maker-chr15-augustus-gene-156.16	yes|up	1
OV10026313.g	maker-chr1-snap-gene-1050.3	yes|down	1
OV20012961.g	maker-chr2-augustus-gene-173.35	yes|up	1
OV80031738.g	maker-chr8-snap-gene-40.35	yes|down	1
OV20015015.g	maker-chr2-snap-gene-684.0	yes|up	1
OV110030236.g	augustus-chr11-processed-gene-55.20	yes|up	1
OV60005611.g	maker-chr6-snap-gene-315.1	yes|up	1
OV120011459.g	maker-chr12-snap-gene-157.5	yes|up	1
OV20013980.g	maker-chr2-snap-gene-432.0	yes|down	1
OV50018294.g	maker-chr5-snap-gene-77.0	yes|down	1
OV10026887.g	maker-chr1-snap-gene-1206.9	yes|down	1
OV10024440.g	maker-chr1-snap-gene-589.7	yes|up	1
OV70020492.g	maker-chr7-snap-gene-99.22	yes|down	1
OV20014886.g	maker-chr2-snap-gene-653.16	yes|up	1
OV140016689.g	maker-chr14-snap-gene-5.28	yes|down	1
OV20012702.g	snap-chr2-processed-gene-114.23	yes|up	1
OV70020597.g	maker-chr7-augustus-gene-119.12	yes|up	1

OV40003390.g	maker-chr4-augustus-gene-446.0	yes|up	1
OV90000748.g	maker-chr9-snap-gene-155.17	yes|down	1
OV20014958.g	maker-chr2-snap-gene-672.42	yes|up	1
OV20013962.g	maker-chr2-snap-gene-426.46	yes|down	1
OV10025520.g	maker-chr1-augustus-gene-862.11	yes|up	1
OV50019843.g	maker-chr5-augustus-gene-426.26	yes|up	1
OV20013573.g	maker-chr2-snap-gene-330.46	yes|down	1
OV100027568.g	maker-chr10-snap-gene-17.55	yes|down	1
OV150028869.g	maker-chr15-snap-gene-14.0	yes|up	1
OV130006401.g	maker-chr13-snap-gene-84.8	yes|down	1
OV30008780.g	maker-chr3-augustus-gene-336.0	yes|down	1
OV80033210.g	maker-chr8-snap-gene-341.52	yes|up	1
OV70021483.g	maker-chr7-exonerate_protein2genome-gene-306.6	yes|up	1
OV50019504.g	maker-chr5-exonerate_est2genome-gene-352.4	yes|up	1
OV90000864.g	maker-chr9-snap-gene-190.13	yes|down	1
OV100028428.g	maker-chr10-exonerate_est2genome-gene-241.51	yes|down	1
OV110030274.g	maker-chr11-snap-gene-62.8	yes|up	1
OV70020676.g	maker-chr7-augustus-gene-136.3	yes|down	1
OV140016778.g	maker-chr14-snap-gene-21.43	yes|up	1
OV20016302.g	maker-chr2-snap-gene-992.9	yes|up	1
OV40002894.g	augustus-chr4-processed-gene-330.33	yes|down	1
OV40002629.g	maker-chr4-augustus-gene-268.3	yes|up	1
OV100028438.g	maker-chr10-exonerate_est2genome-gene-242.21	yes|down	1

OV50018196.g	maker-chr5-augustus-gene-57.12	yes|down	1
OV50018144.g	maker-chr5-augustus-gene-41.51	yes|down	1
OV60004662.g	maker-chr6-snap-gene-96.18	yes|down	1
OV80032803.g	maker-chr8-exonerate_protein2genome-gene-262.0	yes|up	1
OV30008648.g	maker-chr3-augustus-gene-302.0	yes|down	1
OV20014666.g	maker-chr2-snap-gene-593.6	yes|down	1
OV20015844.g	maker-chr2-augustus-gene-881.39	yes|down	1
OV10025443.g	maker-chr1-exonerate_protein2genome-gene-837.2	yes|down	1
OV10024095.g	maker-chr1-augustus-gene-496.3	yes|down	1
OV60004804.g	maker-chr6-augustus-gene-131.33	yes|down	1
OV110030208.g	maker-chr11-snap-gene-48.30	yes|down	1
OV20014664.g	maker-chr2-augustus-gene-593.7	yes|up	1
OV10025441.g	maker-chr1-exonerate_protein2genome-gene-837.0	yes|down	1
OV90000788.g	maker-chr9-exonerate_est2genome-gene-169.1	yes|down	1
OV20015423.g	maker-chr2-augustus-gene-778.15	yes|up	1
OV130006951.g	maker-chr13-exonerate_est2genome-gene-207.2	yes|down	1
OV10024998.g	maker-chr1-snap-gene-729.7	yes|down	1
OV60004384.g	maker-chr6-snap-gene-30.2	yes|down	1
OV10025620.g	maker-chr1-augustus-gene-887.2	yes|down	1
OV30008703.g	maker-chr3-augustus-gene-319.0	yes|up	1
OV40003704.g	maker-chr4-snap-gene-516.4	yes|down	1
OV20016033.g	maker-chr2-snap-gene-925.55	yes|down	1
OV20013466.g	maker-chr2-snap-gene-297.6	yes|up	1

OV150029854.g	maker-chr15-snap-gene-222.3	yes|down	1
OV150029844.g	maker-chr15-snap-gene-220.2	yes|down	1
OV70020367.g	maker-chr7-snap-gene-64.1	yes|down	1
OV80031922.g	maker-chr8-snap-gene-72.0	yes|down	1
OV100028468.g	maker-chr10-snap-gene-250.5	yes|up	1
OV50018770.g	maker-chr5-snap-gene-187.37	yes|down	1
OV70020554.g	maker-chr7-snap-gene-110.12	yes|down	1
OV10023393.g	maker-chr1-snap-gene-306.16	yes|down	1
OV30009895.g	maker-chr3-snap-gene-601.22	yes|down	1
OV80032209.g	maker-chr8-snap-gene-134.57	yes|up	1
OV80033011.g	maker-chr8-snap-gene-298.10	yes|up	1
OV30010629.g	maker-chr3-augustus-gene-782.4	yes|down	1
OV100028011.g	maker-chr10-augustus-gene-128.13	yes|up	1
OV90001556.g	maker-chr9-snap-gene-341.85	yes|down	1
OV120011914.g	maker-chr12-augustus-gene-261.39	yes|up	1
OV50018880.g	maker-chr5-augustus-gene-211.11	yes|down	1
OV110030218.g	maker-chr11-augustus-gene-50.34	yes|down	1
OV80033039.g	maker-chr8-augustus-gene-305.4	yes|down	1
OV40003881.g	maker-chr4-snap-gene-558.3	yes|down	1
OV30007834.g	maker-chr3-snap-gene-106.2	yes|up	1
OV80033013.g	maker-chr8-snap-gene-298.12	yes|down	1
OV50018272.g	augustus-chr5-processed-gene-73.26	yes|up	1
OV20014504.g	maker-chr2-snap-gene-556.44	yes|down	1

OV40002557.g	maker-chr4-snap-gene-249.17	yes|up	1
OV10025415.g	augustus-chr1-processed-gene-827.1	yes|down	1
OV10024512.g	maker-chr1-snap-gene-606.17	yes|down	1
OV70020416.g	maker-chr7-snap-gene-78.22	yes|up	1
OV50018360.g	maker-chr5-snap-gene-97.7	yes|up	1
OV60004449.g	maker-chr6-augustus-gene-50.16	yes|down	1
OV140017627.g	maker-chr14-augustus-gene-216.29	yes|down	1
OV110031085.g	maker-chr11-snap-gene-245.59	yes|up	1
OV120011124.g	augustus-chr12-processed-gene-75.11	yes|down	1
OV20015356.g	maker-chr2-snap-gene-761.42	yes|up	1
OV60004394.g	maker-chr6-augustus-gene-33.11	yes|down	1
OV140016685.g	maker-chr14-snap-gene-5.25	yes|down	1
OV140017500.g	maker-chr14-exonerate_est2genome-gene-192.40	yes|up	1
OV90000716.g	maker-chr9-snap-gene-148.29	yes|down	1
OV10023494.g	maker-chr1-exonerate_protein2genome-gene-329.2	yes|up	1
OV100028545.g	maker-chr10-snap-gene-270.7	yes|down	1
OV10025421.g	maker-chr1-augustus-gene-829.33	yes|down	1
OV110030764.g	maker-chr11-snap-gene-178.36	yes|down	1
OV60005079.g	maker-chr6-snap-gene-196.6	yes|up	1
OV20016553.g	maker-chr2-snap-gene-1057.1	yes|down	1
OV10027294.g	maker-chr1-augustus-gene-1305.30	yes|up	1
OV40001957.g	augustus-chr4-processed-gene-85.5	yes|up	1
OV20012837.g	maker-chr2-snap-gene-147.16	yes|up	1

OV80031965.g	maker-chr8-snap-gene-83.38	yes|down	1
OV20012861.g	maker-chr2-snap-gene-151.11	yes|down	1
OV20016565.g	maker-chr2-exonerate_est2genome-gene-1060.33	yes|up	1
OV150029866.g	maker-chr15-snap-gene-224.9	yes|down	1
OV90000866.g	maker-chr9-exonerate_est2genome-gene-190.19	yes|down	1
OV10022326.g	maker-chr1-snap-gene-53.4	yes|down	1
OV30007493.g	snap-chr3-processed-gene-30.3	yes|down	1
OV120012143.g	maker-chr12-snap-gene-305.5	yes|up	1
OV70020420.g	maker-chr7-snap-gene-78.24	yes|up	1
OV20012960.g	maker-chr2-snap-gene-173.39	yes|up	1
OV10023924.g	maker-chr1-exonerate_est2genome-gene-446.7	yes|down	1
OV150029722.g	maker-chr15-snap-gene-191.32	yes|down	1
OV120011554.g	maker-chr12-exonerate_protein2genome-gene-177.33	yes|up	1
OV30008743.g	maker-chr3-snap-gene-326.10	yes|up	1
OV110030639.g	maker-chr11-snap-gene-151.37	yes|up	1
OV10026178.g	maker-chr1-exonerate_protein2genome-gene-1016.22	yes|up	1
OV10025998.g	maker-chr1-snap-gene-979.34	yes|down	1
OV80032277.g	maker-chr8-snap-gene-149.10	yes|up	1
OV100028666.g	maker-chr10-snap-gene-298.2	yes|down	1
OV80032271.g	maker-chr8-augustus-gene-148.3	yes|up	1
OV60005764.g	maker-chr6-augustus-gene-355.1	yes|up	1
OV10025811.g	maker-chr1-augustus-gene-931.4	yes|up	1

OV40002965.g	maker-chr4-snap-gene-349.1	yes|up	1
OV10027096.g	maker-chr1-snap-gene-1260.24	yes|down	1
OV80031639.g	maker-chr8-snap-gene-19.3	yes|up	1
OV20015279.g	maker-chr2-augustus-gene-741.3	yes|down	1
OV70021527.g	maker-chr7-snap-gene-317.19	yes|down	1
OV80031875.g	maker-chr8-snap-gene-64.16	yes|up	1
OV40004074.g	maker-chr4-snap-gene-598.2	yes|down	1
OV140017264.g	snap-chr14-processed-gene-135.40	yes|down	1
OV90000571.g	maker-chr9-augustus-gene-113.3	yes|down	1
OV30008661.g	maker-chr3-augustus-gene-304.49	yes|down	1
OV60004998.g	maker-chr6-snap-gene-177.20	yes|up	1
OV30007753.g	snap-chr3-processed-gene-84.57	yes|up	1
OV60005875.g	snap-chr6-processed-gene-382.12	yes|down	1
OV140016758.g	maker-chr14-snap-gene-17.26	yes|down	1
OV140016697.g	maker-chr14-snap-gene-7.13	yes|down	1
OV90000171.g	maker-chr9-snap-gene-28.23	yes|down	1
OV30009144.g	maker-chr3-snap-gene-433.6	yes|down	1
OV50018067.g	maker-chr5-snap-gene-26.23	yes|up	1
OV140017461.g	maker-chr14-snap-gene-185.42	yes|down	1
OV30009917.g	maker-chr3-snap-gene-605.42	yes|up	1
OV40002215.g	maker-chr4-augustus-gene-148.2	yes|up	1
OV30010139.g	maker-chr3-augustus-gene-657.49	yes|down	1
OV30009541.g	maker-chr3-snap-gene-522.52	yes|down	1

OV10023266.g	maker-chr1-snap-gene-274.3	yes|down	1
OV20013974.g	snap-chr2-processed-gene-429.4	yes|up	1
OV80032733.g	maker-chr8-snap-gene-246.7	yes|up	1
OV140016868.g	maker-chr14-augustus-gene-36.21	yes|down	1
OV120011098.g	augustus-chr12-processed-gene-71.23	yes|down	1
OV30010631.g	maker-chr3-snap-gene-782.10	yes|down	1
OV120011394.g	maker-chr12-snap-gene-142.1	yes|down	1
OV20014171.g	maker-chr2-exonerate_protein2genome-gene-476.5	yes|down	1
OV140017583.g	maker-chr14-augustus-gene-209.33	yes|up	1
OV10022479.g	maker-chr1-augustus-gene-92.5	yes|down	1
OV110031195.g	maker-chr11-augustus-gene-256.6	yes|down	1
OV90000860.g	maker-chr9-snap-gene-190.11	yes|down	1
OV150029762.g	maker-chr15-snap-gene-203.44	yes|down	1
OV60005263.g	snap-chr6-processed-gene-245.44	yes|up	1
OV20012390.g	augustus-chr2-processed-gene-38.41	yes|up	1
OV30009543.g	maker-chr3-snap-gene-522.53	yes|down	1
OV90000458.g	maker-chr9-snap-gene-84.10	yes|up	1
OV50018768.g	maker-chr5-snap-gene-186.10	yes|down	1
OV20013592.g	maker-chr2-snap-gene-335.11	yes|down	1
OV90001487.g	maker-chr9-snap-gene-322.19	yes|down	1
OV140017464.g	maker-chr14-snap-gene-186.46	yes|down	1
OV10022554.g	maker-chr1-exonerate_protein2genome-gene-110.6	yes|down	1
OV10027451.g	maker-chr1-snap-gene-1340.56	yes|down	1

OV50018824.g	maker-chr5-augustus-gene-196.1	yes|down	1
OV140016937.g	maker-chr14-snap-gene-46.39	yes|up	1
OV100027488.g	maker-chr10-exonerate_est2genome-gene-1.69	yes|up	1
OV50020018.g	maker-chr5-snap-gene-465.27	yes|up	1
OV20014743.g	maker-chr2-snap-gene-615.55	yes|down	1
OV30009925.g	maker-chr3-augustus-gene-607.0	yes|down	1
OV50019003.g	maker-chr5-snap-gene-239.0	yes|up	1
OV80033209.g	maker-chr8-snap-gene-340.16	yes|down	1
OV10024543.g	maker-chr1-exonerate_est2genome-gene-612.2	yes|up	1
OV60005229.g	maker-chr6-snap-gene-235.1	yes|up	1
OV30010128.g	maker-chr3-snap-gene-655.45	yes|up	1
OV50019371.g	maker-chr5-snap-gene-323.56	yes|down	1
OV130006886.g	maker-chr13-snap-gene-196.69	yes|down	1
OV20015214.g	maker-chr2-snap-gene-730.46	yes|up	1
OV50019333.g	maker-chr5-augustus-gene-318.40	yes|down	1
OV40002049.g	maker-chr4-snap-gene-108.0	yes|down	1
OV10025855.g	maker-chr1-augustus-gene-944.2	yes|down	1
OV70021034.g	maker-chr7-snap-gene-212.13	yes|down	1
OV80032741.g	maker-chr8-augustus-gene-249.7	yes|down	1
OV10027453.g	maker-chr1-snap-gene-1341.52	yes|down	1
OV10022598.g	maker-chr1-snap-gene-121.16	yes|down	1
OV60006012.g	maker-chr6-snap-gene-410.4	yes|up	1
OV120011926.g	maker-chr12-exonerate_est2genome-gene-263.3	yes|down	1

OV10027025.g	maker-chr1-snap-gene-1240.5	yes|up	1
OV150029734.g	maker-chr15-snap-gene-193.19	yes|down	1
OV140017422.g	maker-chr14-snap-gene-172.3	yes|down	1
OV30009136.g	maker-chr3-augustus-gene-431.0	yes|up	1
OV150029683.g	maker-chr15-snap-gene-181.31	yes|down	1
OV10027449.g	maker-chr1-snap-gene-1340.55	yes|down	1
OV20015883.g	maker-chr2-augustus-gene-889.0	yes|up	1
OV20013869.g	maker-chr2-snap-gene-398.42	yes|down	1
OV50018170.g	maker-chr5-augustus-gene-52.1	yes|down	1
OV80031844.g	maker-chr8-snap-gene-59.23	yes|up	1
OV20014478.g	maker-chr2-augustus-gene-551.0	yes|down	1
OV150029637.g	maker-chr15-augustus-gene-173.51	yes|up	1
OV120011044.g	maker-chr12-snap-gene-57.0	yes|up	1
OV30008257.g	maker-chr3-snap-gene-203.38	yes|down	1
OV50019795.g	maker-chr5-snap-gene-418.37	yes|up	1
OV90000486.g	maker-chr9-snap-gene-92.37	yes|down	1
OV50018921.g	maker-chr5-exonerate_protein2genome-gene-216.34	yes|up	1
OV60005674.g	maker-chr6-exonerate_protein2genome-gene-330.45	yes|up	1
OV70020831.g	maker-chr7-snap-gene-162.84	yes|up	1
OV60005449.g	maker-chr6-snap-gene-277.4	yes|up	1
OV10025695.g	maker-chr1-augustus-gene-908.7	yes|down	1
OV80032926.g	maker-chr8-snap-gene-280.140	yes|down	1

OV20014002.g	maker-chr2-augustus-gene-439.27	yes|up	1
OV30008164.g	maker-chr3-snap-gene-183.5	yes|up	1
OV120011617.g	maker-chr12-snap-gene-192.3	yes|up	1
OV10023544.g	maker-chr1-snap-gene-348.6	yes|up	1
OV50019429.g	maker-chr5-augustus-gene-338.7	yes|down	1
OV10024725.g	maker-chr1-snap-gene-661.2	yes|up	1
OV80031712.g	maker-chr8-augustus-gene-36.33	yes|down	1
OV60005700.g	maker-chr6-snap-gene-338.10	yes|up	1
OV10026401.g	maker-chr1-snap-gene-1077.0	yes|up	1
OV30009192.g	maker-chr3-snap-gene-444.4	yes|down	1
OV30009152.g	maker-chr3-snap-gene-434.58	yes|down	1
OV130006719.g	maker-chr13-augustus-gene-154.30	yes|down	1
OV20016088.g	maker-chr2-snap-gene-938.63	yes|down	1
OV40003086.g	maker-chr4-augustus-gene-377.0	yes|up	1
OV40002929.g	maker-chr4-snap-gene-337.9	yes|up	1
OV60004300.g	maker-chr6-snap-gene-11.8	yes|up	1
OV20015385.g	maker-chr2-snap-gene-767.57	yes|down	1
OV140017073.g	maker-chr14-snap-gene-84.12	yes|down	1
OV150029292.g	maker-chr15-snap-gene-96.0	yes|up	1
OV30007372.g	maker-chr3-snap-gene-5.6	yes|up	1
OV40003918.g	maker-chr4-snap-gene-567.2	yes|up	1
OV110031504.g	maker-chr11-exonerate_protein2genome-gene-312.28	yes|up	1
OV30010661.g	maker-chr3-augustus-gene-791.20	yes|down	1

OV20016528.g	maker-chr2-snap-gene-1051.0	yes|down	1
OV40004090.g	snap-chr4-processed-gene-602.15	yes|down	1
OV140017462.g	maker-chr14-exonerate_est2genome-gene-186.50	yes|down	1
OV140017107.g	maker-chr14-snap-gene-90.61	yes|down	1
OV130006394.g	maker-chr13-snap-gene-83.73	yes|down	1
OV80031797.g	maker-chr8-augustus-gene-51.23	yes|down	1
OV50019092.g	maker-chr5-augustus-gene-258.39	yes|up	1
OV50019787.g	maker-chr5-augustus-gene-417.5	yes|up	1
OV30009150.g	maker-chr3-snap-gene-434.57	yes|down	1
OV110030828.g	maker-chr11-snap-gene-190.19	yes|down	1
OV50018933.g	maker-chr5-snap-gene-220.18	yes|down	1
OV150029064.g	maker-chr15-snap-gene-57.0	yes|down	1
OV90001536.g	maker-chr9-snap-gene-337.8	yes|up	1
OV40003352.g	maker-chr4-augustus-gene-436.8	yes|up	1
OV10025493.g	augustus-chr1-processed-gene-854.12	yes|down	1
OV100027562.g	maker-chr10-augustus-gene-16.14	yes|down	1
OV60004918.g	maker-chr6-augustus-gene-156.34	yes|down	1
OV80032448.g	maker-chr8-snap-gene-183.26	yes|up	1
OV40003167.g	maker-chr4-snap-gene-394.19	yes|up	1
OV120011062.g	maker-chr12-snap-gene-62.2	yes|down	1
OV60004922.g	maker-chr6-snap-gene-156.5	yes|down	1
OV10024321.g	maker-chr1-snap-gene-556.0	yes|up	1
OV20015165.g	maker-chr2-snap-gene-718.21	yes|down	1

OV80031793.g	augustus-chr8-processed-gene-50.3	yes|down	1
OV100028264.g	maker-chr10-augustus-gene-191.19	yes|up	1
OV120012126.g	maker-chr12-exonerate_protein2genome-gene-300.8	yes|up	1
OV150028883.g	maker-chr15-exonerate_est2genome-gene-17.24	yes|down	1
OV140016882.g	maker-chr14-snap-gene-38.16	yes|down	1
OV30009763.g	maker-chr3-snap-gene-570.0	yes|up	1
OV150029836.g	maker-chr15-augustus-gene-218.34	yes|down	1
OV10023050.g	maker-chr1-snap-gene-222.30	yes|down	1
OV40003153.g	maker-chr4-exonerate_protein2genome-gene-390.20	yes|down	1
OV30010548.g	maker-chr3-snap-gene-761.84	yes|up	1
OV30008542.g	maker-chr3-snap-gene-271.44	yes|down	1
OV60005766.g	maker-chr6-snap-gene-356.41	yes|down	1
OV130007058.g	maker-chr13-exonerate_protein2genome-gene-235.17	yes|down	1
OV50019868.g	maker-chr5-snap-gene-435.42	yes|up	1
OV130006606.g	maker-chr13-snap-gene-123.24	yes|down	1
OV60004689.g	maker-chr6-snap-gene-102.4	yes|up	1
OV30008872.g	maker-chr3-augustus-gene-358.30	yes|up	1
OV100028535.g	maker-chr10-snap-gene-269.6	yes|down	1
OV110030594.g	maker-chr11-snap-gene-141.28	yes|up	1
OV90000240.g	maker-chr9-exonerate_est2genome-gene-36.3	yes|down	1
OV40001975.g	maker-chr4-snap-gene-88.10	yes|up	1
OV120010897.g	maker-chr12-snap-gene-13.41	yes|down	1

OV10022408.g	maker-chr1-augustus-gene-75.59	yes|down	1
OV90000597.g	maker-chr9-augustus-gene-122.23	yes|down	1
OV70021460.g	maker-chr7-snap-gene-299.17	yes|up	1
OV90001483.g	maker-chr9-snap-gene-322.17	yes|down	1
OV10027184.g	maker-chr1-snap-gene-1279.32	yes|down	1
OV10027316.g	maker-chr1-augustus-gene-1309.33	yes|up	1
OV150029708.g	maker-chr15-augustus-gene-185.32	yes|down	1
OV40003761.g	maker-chr4-augustus-gene-531.28	yes|down	1
OV50018791.g	maker-chr5-augustus-gene-192.6	yes|up	1
OV20015208.g	maker-chr2-snap-gene-728.12	yes|down	1
OV40003267.g	maker-chr4-snap-gene-418.43	yes|up	1
OV80032424.g	maker-chr8-snap-gene-177.21	yes|up	1
OV60004516.g	maker-chr6-augustus-gene-62.104	yes|down	1
OV20016167.g	maker-chr2-exonerate_protein2genome-gene-959.45	yes|up	1
OV20014479.g	snap-chr2-processed-gene-552.6	yes|up	1
OV10024922.g	maker-chr1-augustus-gene-712.0	yes|down	1
OV30010791.g	maker-chr3-snap-gene-820.55	yes|down	1
OV110030414.g	maker-chr11-snap-gene-96.17	yes|down	1
OV110030527.g	maker-chr11-snap-gene-126.15	yes|up	1
OV10027182.g	maker-chr1-snap-gene-1279.33	yes|down	1
OV10023556.g	maker-chr1-snap-gene-351.4	yes|up	1
OV120011270.g	maker-chr12-snap-gene-110.25	yes|up	1

OV30007592.g	maker-chr3-augustus-gene-51.2	yes|down	1
OV30009868.g	augustus-chr3-processed-gene-593.16	yes|up	1
OV10024775.g	maker-chr1-snap-gene-672.25	yes|down	1
OV50019315.g	maker-chr5-snap-gene-312.20	yes|down	1
OV30010040.g	maker-chr3-exonerate_protein2genome-gene-632.10	yes|up	1
OV70021412.g	maker-chr7-augustus-gene-288.1	yes|down	1
OV20012239.g	maker-chr2-augustus-gene-7.49	yes|down	1
OV10023085.g	maker-chr1-snap-gene-232.10	yes|down	1
OV20012336.g	maker-chr2-snap-gene-26.0	yes|down	1
OV30008124.g	maker-chr3-snap-gene-170.4	yes|down	1
OV110031477.g	maker-chr11-snap-gene-305.48	yes|up	1
OV80032800.g	snap-chr8-processed-gene-260.1	yes|down	1
OV40001898.g	maker-chr4-augustus-gene-69.1	yes|down	1
OV40003709.g	maker-chr4-snap-gene-518.36	yes|down	1
OV110030951.g	maker-chr11-snap-gene-213.51	yes|down	1
OV90001407.g	maker-chr9-snap-gene-306.81	yes|down	1
OV20012225.g	augustus-chr2-processed-gene-5.11	yes|up	1
OV20015641.g	maker-chr2-snap-gene-837.3	yes|up	1
OV70021567.g	maker-chr7-exonerate_est2genome-gene-323.50	yes|down	1
OV80033115.g	maker-chr8-snap-gene-322.0	yes|up	1
OV20014118.g	maker-chr2-augustus-gene-466.1	yes|down	1
OV30007618.g	maker-chr3-snap-gene-56.24	yes|down	1
OV70020979.g	maker-chr7-snap-gene-197.3	yes|down	1

OV40004023.g	maker-chr4-augustus-gene-588.20	yes|up	1
OV20016016.g	maker-chr2-snap-gene-922.2	yes|up	1
OV50018566.g	maker-chr5-augustus-gene-141.18	yes|down	1
OV30008530.g	maker-chr3-augustus-gene-266.33	yes|up	1
OV130007247.g	maker-chr13-snap-gene-284.8	yes|up	1
OV20016339.g	maker-chr2-exonerate_protein2genome-gene-1000.5	yes|down	1
OV60004491.g	maker-chr6-snap-gene-56.27	yes|down	1
OV20015573.g	maker-chr2-snap-gene-819.0	yes|up	1
OV100028232.g	maker-chr10-snap-gene-185.31	yes|up	1
OV110030947.g	maker-chr11-augustus-gene-213.0	yes|up	1
OV30009856.g	maker-chr3-snap-gene-590.2	yes|up	1
OV130007241.g	maker-chr13-snap-gene-282.2	yes|down	1
OV110030872.g	maker-chr11-snap-gene-196.2	yes|down	1
OV130007052.g	maker-chr13-snap-gene-234.8	yes|down	1
OV120011066.g	maker-chr12-snap-gene-63.25	yes|up	1
OV30008228.g	maker-chr3-augustus-gene-200.37	yes|down	1
OV20014078.g	maker-chr2-snap-gene-455.7	yes|down	1
OV70021177.g	maker-chr7-augustus-gene-242.6	yes|down	1
OV90001419.g	maker-chr9-augustus-gene-308.9	yes|down	1
OV70021769.g	maker-chr7-augustus-gene-353.0	yes|up	1
OV50018398.g	maker-chr5-snap-gene-105.13	yes|up	1
OV30010160.g	maker-chr3-augustus-gene-662.19	yes|down	1

OV60004736.g	maker-chr6-snap-gene-110.19	yes|up	1
OV20015950.g	maker-chr2-augustus-gene-904.24	yes|up	1
OV40003368.g	maker-chr4-augustus-gene-441.61	yes|up	1
OV30009840.g	maker-chr3-snap-gene-587.1	yes|up	1
OV110031294.g	maker-chr11-exonerate_est2genome-gene-271.90	yes|down	1
OV20014999.g	maker-chr2-augustus-gene-681.5	yes|down	1
OV20016256.g	maker-chr2-augustus-gene-980.17	yes|up	1
OV140016823.g	maker-chr14-snap-gene-31.22	yes|down	1
OV40002375.g	maker-chr4-snap-gene-191.12	yes|up	1
OV10022539.g	maker-chr1-exonerate_protein2genome-gene-106.11	yes|up	1
OV110031028.g	augustus-chr11-processed-gene-233.1	yes|down	1
OV10025618.g	maker-chr1-augustus-gene-885.3	yes|down	1
OV40003366.g	maker-chr4-snap-gene-440.7	yes|up	1
OV50019989.g	maker-chr5-snap-gene-458.9	yes|down	1
OV20015010.g	maker-chr2-augustus-gene-682.14	yes|up	1
OV150028979.g	maker-chr15-snap-gene-32.24	yes|down	1
OV60004298.g	maker-chr6-augustus-gene-9.28	yes|up	1
OV60005523.g	maker-chr6-exonerate_est2genome-gene-294.1	yes|up	1
OV120011179.g	maker-chr12-snap-gene-89.11	yes|down	1
OV10023006.g	maker-chr1-exonerate_protein2genome-gene-212.15	yes|down	1
OV20013903.g	maker-chr2-augustus-gene-412.1	yes|up	1
OV30008779.g	maker-chr3-exonerate_protein2genome-gene-336.8	yes|down	1

OV110030963.g	maker-chr11-snap-gene-216.46	yes|up	1
OV150029704.g	maker-chr15-snap-gene-184.4	yes|up	1
OV20014137.g	maker-chr2-exonerate_protein2genome-gene-471.7	yes|down	1
OV90001236.g	maker-chr9-snap-gene-277.44	yes|up	1
OV30009790.g	maker-chr3-augustus-gene-576.17	yes|down	1
OV80031953.g	maker-chr8-snap-gene-80.3	yes|down	1
OV70022032.g	maker-chr7-augustus-gene-402.17	yes|down	1
OV10025655.g	maker-chr1-augustus-gene-896.12	yes|down	1
OV20015882.g	maker-chr2-snap-gene-888.44	yes|up	1
OV60004748.g	maker-chr6-augustus-gene-114.33	yes|down	1
OV130006644.g	maker-chr13-exonerate_est2genome-gene-133.16	yes|down	1
OV20015056.g	maker-chr2-snap-gene-694.10	yes|up	1
OV20012889.g	maker-chr2-snap-gene-155.5	yes|down	1
OV110030126.g	maker-chr11-snap-gene-31.25	yes|down	1
OV30009111.g	maker-chr3-snap-gene-423.7	yes|up	1
OV10026659.g	maker-chr1-snap-gene-1148.31	yes|down	1
OV140017277.g	maker-chr14-snap-gene-140.13	yes|down	1
OV20012428.g	maker-chr2-snap-gene-48.23	yes|up	1
OV130006112.g	maker-chr13-snap-gene-10.3	yes|up	1
OV80032963.g	maker-chr8-snap-gene-284.28	yes|down	1
OV80031731.g	maker-chr8-augustus-gene-39.38	yes|up	1
OV10022124.g	snap-chr1-processed-gene-2.65	yes|down	1
OV10025545.g	maker-chr1-augustus-gene-867.26	yes|up	1

OV150029392.g	maker-chr15-snap-gene-119.16	yes|down	1
OV130007189.g	maker-chr13-augustus-gene-267.70	yes|up	1
OV40003992.g	maker-chr4-snap-gene-582.4	yes|up	1
OV20012696.g	maker-chr2-augustus-gene-113.4	yes|up	1
OV110031471.g	maker-chr11-exonerate_protein2genome-gene-304.43	yes|up	1
OV20016403.g	maker-chr2-exonerate_est2genome-gene-1016.2	yes|up	1
OV40002647.g	maker-chr4-snap-gene-271.19	yes|up	1
OV60005108.g	maker-chr6-augustus-gene-205.0	yes|up	1
OV40003707.g	maker-chr4-augustus-gene-517.12	yes|down	1
OV40002149.g	maker-chr4-exonerate_est2genome-gene-132.5	yes|down	1
OV140017517.g	maker-chr14-augustus-gene-195.7	yes|down	1
OV120011828.g	maker-chr12-augustus-gene-240.3	yes|up	1
OV90001147.g	maker-chr9-exonerate_est2genome-gene-255.39	yes|down	1
OV110031425.g	maker-chr11-augustus-gene-296.0	yes|up	1
OV10024412.g	maker-chr1-snap-gene-583.40	yes|up	1
OV50018157.g	augustus-chr5-processed-gene-48.0	yes|up	1
OV20012787.g	maker-chr2-snap-gene-135.23	yes|down	1
OV20016550.g	maker-chr2-snap-gene-1057.2	yes|down	1
OV110030763.g	maker-chr11-snap-gene-178.35	yes|down	1
OV10023300.g	maker-chr1-augustus-gene-281.33	yes|up	1
OV20013965.g	maker-chr2-augustus-gene-427.1	yes|up	1
OV30007796.g	maker-chr3-snap-gene-96.16	yes|up	1
OV10023402.g	maker-chr1-snap-gene-309.6	yes|up	1

OV20015871.g	maker-chr2-snap-gene-887.8	yes|up	1
OV10024414.g	maker-chr1-snap-gene-583.41	yes|up	1
OV40004069.g	maker-chr4-snap-gene-597.25	yes|down	1
OV50019738.g	maker-chr5-snap-gene-399.36	yes|up	1
OV50018904.g	snap-chr5-processed-gene-215.1	yes|up	1
OV10026736.g	maker-chr1-augustus-gene-1167.34	yes|down	1
OV140017789.g	maker-chr14-augustus-gene-248.0	yes|up	1
OV80032907.g	maker-chr8-exonerate_protein2genome-gene-278.44	yes|up	1
OV10025671.g	maker-chr1-augustus-gene-901.7	yes|down	1
OV50019123.g	maker-chr5-augustus-gene-268.14	yes|up	1
OV100028597.g	maker-chr10-snap-gene-283.31	yes|down	1
OV90000614.g	maker-chr9-snap-gene-127.1	yes|up	1
OV80031735.g	maker-chr8-snap-gene-39.5	yes|up	1
OV60005800.g	maker-chr6-snap-gene-363.2	yes|down	1
OV50018154.g	maker-chr5-snap-gene-48.3	yes|up	1
OV10022648.g	snap-chr1-processed-gene-130.6	yes|down	1
OV10022668.g	maker-chr1-augustus-gene-134.9	yes|down	1
OV140017155.g	maker-chr14-augustus-gene-105.24	yes|up	1
OV40003944.g	maker-chr4-augustus-gene-574.4	yes|down	1
OV120010938.g	maker-chr12-snap-gene-28.21	yes|down	1
OV10027176.g	maker-chr1-snap-gene-1278.31	yes|down	1
OV40003581.g	maker-chr4-snap-gene-489.21	yes|down	1

OV20016630.g	maker-chr2-snap-gene-1078.1	yes|down	1
OV30007537.g	maker-chr3-augustus-gene-38.15	yes|down	1
OV10023847.g	maker-chr1-snap-gene-426.13	yes|down	1
OV20014359.g	maker-chr2-snap-gene-519.31	yes|down	1
OV50018302.g	maker-chr5-snap-gene-79.25	yes|down	1
OV150029103.g	maker-chr15-augustus-gene-63.2	yes|down	1
OV70021089.g	snap-chr7-processed-gene-224.28	yes|up	1
OV100028043.g	maker-chr10-snap-gene-138.12	yes|up	1
OV40002090.g	maker-chr4-snap-gene-121.5	yes|up	1
OV50019191.g	maker-chr5-snap-gene-286.37	yes|down	1
OV80031964.g	maker-chr8-snap-gene-83.37	yes|up	1
OV20016636.g	maker-chr2-snap-gene-1081.9	yes|up	1
OV120011029.g	maker-chr12-snap-gene-54.44	yes|down	1
OV130006897.g	maker-chr13-snap-gene-198.38	yes|down	1
OV20014953.g	maker-chr2-snap-gene-671.31	yes|down	1
OV30009944.g	maker-chr3-snap-gene-611.32	yes|up	1
	OV130006146.g	yes|up	1
OV150028894.g	maker-chr15-exonerate_est2genome-gene-18.2	yes|up	1
OV70020455.g	maker-chr7-snap-gene-89.40	yes|up	1
OV70021138.g	maker-chr7-snap-gene-234.38	yes|down	1
OV30008839.g	maker-chr3-augustus-gene-350.2	yes|up	1
OV30007713.g	maker-chr3-augustus-gene-79.12	yes|down	1
OV110030953.g	maker-chr11-snap-gene-213.52	yes|down	1

OV60005531.g	maker-chr6-augustus-gene-296.3	yes|down	1
OV40002421.g	maker-chr4-augustus-gene-206.33	yes|down	1
OV140016825.g	maker-chr14-snap-gene-31.24	yes|down	1
OV40003921.g	maker-chr4-snap-gene-567.0	yes|up	1
OV10025612.g	maker-chr1-snap-gene-882.3	yes|down	1
OV30008645.g	maker-chr3-snap-gene-300.1	yes|down	1
OV10024734.g	maker-chr1-exonerate_est2genome-gene-662.16	yes|down	1
OV110030095.g	maker-chr11-snap-gene-25.9	yes|down	1
OV70020697.g	maker-chr7-augustus-gene-141.6	yes|down	1
OV20015270.g	maker-chr2-snap-gene-739.18	yes|down	1
OV50018257.g	maker-chr5-snap-gene-70.10	yes|down	1
OV50018084.g	maker-chr5-snap-gene-29.68	yes|down	1
OV130006460.g	maker-chr13-snap-gene-96.39	yes|down	1
OV80031994.g	maker-chr8-augustus-gene-88.18	yes|down	1
OV120010954.g	maker-chr12-snap-gene-33.1	yes|up	1
OV90001268.g	maker-chr9-exonerate_protein2genome-gene-287.6	yes|down	1
OV40002516.g	maker-chr4-augustus-gene-233.20	yes|down	1
OV30008290.g	maker-chr3-snap-gene-210.11	yes|down	1
OV30009022.g	maker-chr3-snap-gene-400.14	yes|down	1
OV130007228.g	maker-chr13-snap-gene-278.63	yes|down	1
OV70021081.g	maker-chr7-snap-gene-222.11	yes|down	1
OV30010300.g	maker-chr3-augustus-gene-702.0	yes|down	1
OV20016548.g	maker-chr2-snap-gene-1056.22	yes|up	1

OV10023876.g	maker-chr1-snap-gene-435.21	yes|up	1
OV10025967.g	maker-chr1-augustus-gene-973.4	yes|down	1
OV70020089.g	maker-chr7-snap-gene-7.11	yes|down	1
OV90000865.g	snap-chr9-processed-gene-190.28	yes|down	1
OV10023406.g	maker-chr1-exonerate_est2genome-gene-309.34	yes|up	1
OV80031660.g	maker-chr8-snap-gene-22.3	yes|down	1
OV50018145.g	maker-chr5-snap-gene-41.45	yes|down	1
OV30009511.g	maker-chr3-augustus-gene-513.44	yes|up	1
OV10022259.g	maker-chr1-snap-gene-34.1	yes|down	1
OV50018377.g	maker-chr5-snap-gene-100.29	yes|down	1
OV20015032.g	maker-chr2-snap-gene-687.4	yes|down	1
OV30009667.g	maker-chr3-snap-gene-550.10	yes|down	1
OV50018153.g	maker-chr5-snap-gene-47.6	yes|up	1
OV90000634.g	maker-chr9-augustus-gene-131.25	yes|down	1
OV60004379.g	snap-chr6-processed-gene-28.18	yes|down	1
OV30009489.g	maker-chr3-snap-gene-506.28	yes|up	1
OV20015516.g	maker-chr2-augustus-gene-803.12	yes|down	1
OV20012438.g	maker-chr2-augustus-gene-50.13	yes|down	1
OV30007813.g	maker-chr3-snap-gene-100.0	yes|up	1
OV140017494.g	maker-chr14-augustus-gene-191.2	yes|down	1
OV20015309.g	maker-chr2-augustus-gene-748.51	yes|down	1
OV40002558.g	maker-chr4-augustus-gene-249.21	yes|up	1
OV70020153.g	augustus-chr7-processed-gene-18.26	yes|down	1

OV20015894.g	maker-chr2-snap-gene-891.14	yes|up	1
OV80033212.g	maker-chr8-snap-gene-341.50	yes|down	1
OV60004513.g	maker-chr6-snap-gene-61.18	yes|down	1
OV40002665.g	maker-chr4-augustus-gene-275.46	yes|up	1
OV30010630.g	maker-chr3-snap-gene-782.9	yes|down	1
OV150029761.g	maker-chr15-snap-gene-203.43	yes|down	1
OV50020017.g	maker-chr5-snap-gene-465.28	yes|down	1
OV10026359.g	maker-chr1-snap-gene-1061.2	yes|up	1
OV10027450.g	maker-chr1-exonerate_est2genome-gene-1340.3	yes|down	1
OV20016380.g	maker-chr2-exonerate_protein2genome-gene-1011.15	yes|up	1
OV90000715.g	snap-chr9-processed-gene-148.10	yes|down	1
OV120011683.g	maker-chr12-augustus-gene-209.2	yes|up	1
OV110030930.g	maker-chr11-augustus-gene-209.1	yes|down	1
OV50018325.g	maker-chr5-exonerate_est2genome-gene-87.59	yes|down	1
OV50018251.g	maker-chr5-augustus-gene-69.48	yes|up	1
OV50019239.g	augustus-chr5-processed-gene-298.2	yes|up	1
OV100027816.g	maker-chr10-augustus-gene-77.37	yes|up	1
OV100028504.g	maker-chr10-snap-gene-261.0	yes|up	1
OV150029117.g	maker-chr15-augustus-gene-66.0	yes|down	1
OV30007829.g	maker-chr3-augustus-gene-103.15	yes|down	1
OV30010752.g	maker-chr3-snap-gene-813.22	yes|down	1
OV90000912.g	maker-chr9-snap-gene-201.20	yes|down	1

OV150029115.g	maker-chr15-augustus-gene-66.5	yes|down	1
OV80032513.g	maker-chr8-augustus-gene-199.60	yes|down	1
OV30010587.g	maker-chr3-exonerate_protein2genome-gene-771.10	yes|down	1
OV90000863.g	maker-chr9-snap-gene-190.12	yes|down	1
OV120011108.g	maker-chr12-augustus-gene-73.0	yes|up	1
OV30009766.g	maker-chr3-augustus-gene-570.26	yes|up	1
OV80033174.g	maker-chr8-snap-gene-332.28	yes|up	1
OV100028014.g	snap-chr10-processed-gene-128.56	yes|up	1
OV70020748.g	maker-chr7-snap-gene-150.31	yes|up	1
OV100027970.g	maker-chr10-snap-gene-115.17	yes|down	1
OV80032108.g	maker-chr8-snap-gene-112.19	yes|down	1
OV30009924.g	maker-chr3-augustus-gene-606.5	yes|down	1
OV10023703.g	maker-chr1-snap-gene-387.19	yes|up	1
OV90000885.g	maker-chr9-snap-gene-196.2	yes|up	1
OV80033152.g	maker-chr8-snap-gene-328.37	yes|down	1
OV60005959.g	maker-chr6-snap-gene-397.1	yes|down	1
OV110031188.g	maker-chr11-snap-gene-254.30	yes|down	1
OV70020568.g	maker-chr7-snap-gene-115.15	yes|down	1
OV90000293.g	maker-chr9-snap-gene-46.0	yes|down	1
OV30009141.g	maker-chr3-snap-gene-432.5	yes|up	1
OV130006254.g	maker-chr13-snap-gene-46.23	yes|down	1
OV10023225.g	maker-chr1-exonerate_protein2genome-gene-266.21	yes|up	1

OV10022368.g	maker-chr1-snap-gene-63.12	yes|down	1
OV10024588.g	maker-chr1-snap-gene-622.30	yes|down	1
OV150029484.g	maker-chr15-snap-gene-142.13	yes|down	1
OV110031192.g	maker-chr11-augustus-gene-255.2	yes|down	1
OV10025350.g	maker-chr1-exonerate_protein2genome-gene-809.6	yes|up	1
OV10022165.g	maker-chr1-augustus-gene-9.5	yes|up	1
OV50018208.g	maker-chr5-augustus-gene-60.31	yes|down	1
OV70022103.g	augustus-chr7-processed-gene-413.23	yes|up	1
OV50019089.g	maker-chr5-snap-gene-258.33	yes|up	1
OV10024925.g	maker-chr1-snap-gene-714.3	yes|down	1
OV110030217.g	maker-chr11-snap-gene-49.41	yes|up	1
OV10023923.g	maker-chr1-augustus-gene-446.15	yes|up	1
OV70020501.g	maker-chr7-snap-gene-100.15	yes|up	1
OV120011628.g	maker-chr12-augustus-gene-196.41	yes|up	1
OV10024576.g	maker-chr1-augustus-gene-620.27	yes|up	1
OV40004071.g	maker-chr4-augustus-gene-598.4	yes|down	1
OV110030415.g	maker-chr11-augustus-gene-96.10	yes|down	1
OV110029984.g	maker-chr11-snap-gene-3.5	yes|down	1
OV80031647.g	augustus-chr8-processed-gene-19.39	yes|down	1
OV150029602.g	maker-chr15-augustus-gene-166.32	yes|down	1
OV30008954.g	maker-chr3-augustus-gene-380.56	yes|down	1
OV10024679.g	maker-chr1-snap-gene-645.5	yes|up	1
OV150028832.g	maker-chr15-augustus-gene-5.26	yes|down	1

OV10024524.g	maker-chr1-augustus-gene-608.25	yes|up	1
OV60004428.g	maker-chr6-snap-gene-46.22	yes|down	1
OV80032956.g	maker-chr8-augustus-gene-283.68	yes|down	1
OV130006732.g	maker-chr13-snap-gene-158.7	yes|up	1
OV110031289.g	maker-chr11-snap-gene-271.78	yes|up	1
OV80032290.g	maker-chr8-exonerate_protein2genome-gene-154.21	yes|down	1
OV30009145.g	maker-chr3-augustus-gene-433.35	yes|down	1
OV30009153.g	maker-chr3-exonerate_protein2genome-gene-434.35	yes|down	1
OV60005654.g	maker-chr6-snap-gene-326.15	yes|down	1
OV100028671.g	maker-chr10-exonerate_est2genome-gene-299.0	yes|up	1
OV100027789.g	maker-chr10-exonerate_est2genome-gene-68.9	yes|down	1
OV40002453.g	maker-chr4-augustus-gene-216.19	yes|up	1
OV20013576.g	maker-chr2-snap-gene-330.48	yes|up	1
OV10026357.g	maker-chr1-snap-gene-1059.6	yes|up	1
OV30009699.g	maker-chr3-snap-gene-558.36	yes|down	1
OV50018214.g	maker-chr5-augustus-gene-62.8	yes|down	1
OV20015384.g	maker-chr2-augustus-gene-767.42	yes|up	1
OV30008240.g	maker-chr3-snap-gene-201.2	yes|up	1
OV50017996.g	maker-chr5-snap-gene-14.11	yes|down	1
OV10026461.g	maker-chr1-snap-gene-1091.5	yes|down	1
OV90000510.g	maker-chr9-snap-gene-98.6	yes|down	1
OV20013486.g	maker-chr2-snap-gene-302.27	yes|up	1

OV150029412.g	maker-chr15-augustus-gene-124.1	yes|down	1
OV10022304.g	maker-chr1-augustus-gene-47.36	yes|down	1
OV20013531.g	maker-chr2-snap-gene-315.31	yes|up	1
OV10027293.g	augustus-chr1-processed-gene-1304.9	yes|up	1
OV70021107.g	maker-chr7-augustus-gene-229.85	yes|up	1
OV70020200.g	maker-chr7-augustus-gene-30.17	yes|down	1
OV30009151.g	maker-chr3-exonerate_protein2genome-gene-434.46	yes|down	1
OV10026351.g	maker-chr1-augustus-gene-1059.43	yes|down	1
OV90000564.g	maker-chr9-snap-gene-112.4	yes|up	1
OV110031186.g	maker-chr11-snap-gene-254.29	yes|down	1
OV140017534.g	maker-chr14-snap-gene-198.0	yes|down	1
OV10024482.g	maker-chr1-exonerate_est2genome-gene-597.10	yes|up	1
OV120012115.g	maker-chr12-snap-gene-299.5	yes|up	1
OV60005699.g	snap-chr6-processed-gene-337.38	yes|up	1
OV10026054.g	maker-chr1-snap-gene-992.9	yes|up	1
OV90000686.g	maker-chr9-augustus-gene-141.2	yes|down	1
OV30009736.g	maker-chr3-snap-gene-565.5	yes|up	1
OV100027754.g	maker-chr10-snap-gene-61.4	yes|down	1
OV10026003.g	augustus-chr1-processed-gene-980.31	yes|up	1
OV60005008.g	maker-chr6-exonerate_est2genome-gene-180.5	yes|down	1
OV60004583.g	maker-chr6-augustus-gene-78.41	yes|up	1
OV30008846.g	maker-chr3-snap-gene-352.12	yes|down	1

OV110030809.g	maker-chr11-snap-gene-187.18	yes|down	1
OV150029080.g	maker-chr15-augustus-gene-61.8	yes|down	1
OV30010551.g	maker-chr3-snap-gene-762.1	yes|up	1
OV100027762.g	maker-chr10-snap-gene-64.7	yes|up	1
OV110030775.g	maker-chr11-snap-gene-180.8	yes|down	1
OV10024587.g	maker-chr1-exonerate_protein2genome-gene-622.12	yes|down	1
OV10026067.g	maker-chr1-snap-gene-996.46	yes|down	1
OV100027643.g	maker-chr10-augustus-gene-39.12	yes|down	1
OV30009092.g	maker-chr3-augustus-gene-419.26	yes|down	1
OV110030837.g	maker-chr11-snap-gene-191.30	yes|up	1
OV90000154.g	maker-chr9-snap-gene-25.20	yes|down	1
OV40004075.g	maker-chr4-augustus-gene-598.5	yes|down	1
OV80032744.g	maker-chr8-augustus-gene-250.14	yes|down	1
OV120011492.g	maker-chr12-snap-gene-162.13	yes|up	1
OV90000685.g	maker-chr9-snap-gene-141.44	yes|down	1
OV60005955.g	maker-chr6-snap-gene-396.21	yes|down	1
OV20012878.g	maker-chr2-exonerate_protein2genome-gene-154.4	yes|up	1
OV30009093.g	augustus-chr3-processed-gene-419.2	yes|up	1
OV90001011.g	maker-chr9-exonerate_est2genome-gene-224.0	yes|up	1
OV70020397.g	maker-chr7-augustus-gene-75.29	yes|down	1
OV40003844.g	maker-chr4-snap-gene-551.39	yes|up	1
OV70020566.g	maker-chr7-snap-gene-114.20	yes|up	1
OV30009704.g	maker-chr3-snap-gene-559.0	yes|up	1

OV90001486.g	maker-chr9-snap-gene-323.19	yes|down	1
OV150029757.g	maker-chr15-snap-gene-201.27	yes|down	1
OV70020396.g	maker-chr7-augustus-gene-75.27	yes|down	1
OV20014055.g	maker-chr2-snap-gene-450.0	yes|down	1
OV20012973.g	maker-chr2-snap-gene-177.13	yes|down	1
OV100027870.g	maker-chr10-augustus-gene-94.1	yes|up	1
OV110030004.g	maker-chr11-snap-gene-5.11	yes|down	1
OV90000407.g	maker-chr9-snap-gene-69.34	yes|down	1
OV70022043.g	maker-chr7-snap-gene-405.64	yes|down	1
OV30007760.g	maker-chr3-snap-gene-86.54	yes|down	1
OV30008970.g	maker-chr3-exonerate_protein2genome-gene-387.0	yes|down	1
OV20015222.g	maker-chr2-augustus-gene-731.10	yes|down	1
OV50019026.g	maker-chr5-snap-gene-243.3	yes|up	1
OV140016693.g	maker-chr14-augustus-gene-6.4	yes|down	1
OV40002928.g	maker-chr4-snap-gene-337.12	yes|up	1
OV90001182.g	maker-chr9-snap-gene-267.34	yes|up	1
OV10024237.g	maker-chr1-augustus-gene-535.45	yes|down	1
OV110031388.g	maker-chr11-augustus-gene-289.4	yes|up	1
OV60005082.g	maker-chr6-augustus-gene-198.17	yes|up	1
OV10022445.g	maker-chr1-snap-gene-83.38	yes|up	1
OV140017143.g	maker-chr14-augustus-gene-103.7	yes|down	1
OV120010884.g	maker-chr12-snap-gene-11.3	yes|down	1
OV150029059.g	augustus-chr15-processed-gene-54.8	yes|up	1

OV70020930.g	maker-chr7-snap-gene-188.18	yes|up	1
OV140016909.g	snap-chr14-processed-gene-44.55	yes|down	1
OV30007754.g	maker-chr3-augustus-gene-85.26	yes|down	1
OV30010108.g	maker-chr3-snap-gene-650.40	yes|up	1
OV30008266.g	maker-chr3-augustus-gene-207.31	yes|down	1
OV20014003.g	maker-chr2-snap-gene-439.22	yes|up	1
OV10023095.g	maker-chr1-augustus-gene-236.1	yes|down	1
OV30008202.g	maker-chr3-snap-gene-194.69	yes|up	1
OV150029721.g	maker-chr15-augustus-gene-191.37	yes|down	1
OV90001547.g	maker-chr9-snap-gene-339.4	yes|up	1
OV20013259.g	maker-chr2-snap-gene-247.28	yes|up	1
OV50017965.g	maker-chr5-snap-gene-7.41	yes|down	1
OV30008529.g	maker-chr3-augustus-gene-266.36	yes|up	1
OV150029037.g	maker-chr15-exonerate_est2genome-gene-48.15	yes|up	1
OV50019541.g	maker-chr5-snap-gene-359.48	yes|up	1
OV60005042.g	maker-chr6-snap-gene-184.18	yes|up	1
OV60004303.g	maker-chr6-snap-gene-11.9	yes|down	1
OV40003578.g	maker-chr4-snap-gene-489.17	yes|down	1
OV110029987.g	maker-chr11-snap-gene-3.3	yes|down	1
OV110029999.g	maker-chr11-snap-gene-5.9	yes|down	1
OV30008115.g	maker-chr3-exonerate_protein2genome-gene-168.14	yes|up	1
OV10024199.g	maker-chr1-augustus-gene-522.17	yes|down	1
OV10023132.g	maker-chr1-snap-gene-246.35	yes|up	1

OV20014318.g	maker-chr2-snap-gene-510.58	yes|down	1
OV80032373.g	maker-chr8-snap-gene-169.14	yes|up	1
OV40002475.g	maker-chr4-snap-gene-222.38	yes|up	1
OV20015872.g	maker-chr2-snap-gene-887.5	yes|up	1
OV110029989.g	maker-chr11-snap-gene-3.4	yes|down	1
OV10025377.g	maker-chr1-augustus-gene-816.30	yes|down	1
OV10026034.g	maker-chr1-snap-gene-986.43	yes|up	1
OV40003800.g	maker-chr4-snap-gene-540.41	yes|up	1
OV110030012.g	maker-chr11-exonerate_est2genome-gene-7.10	yes|up	1
OV60004345.g	maker-chr6-snap-gene-20.43	yes|down	1
OV140017435.g	maker-chr14-snap-gene-179.3	yes|down	1
OV20013731.g	maker-chr2-snap-gene-367.65	yes|down	1
OV110030188.g	maker-chr11-augustus-gene-44.0	yes|down	1
OV20015905.g	maker-chr2-snap-gene-895.7	yes|down	1
OV70021816.g	maker-chr7-snap-gene-362.43	yes|down	1
OV90000489.g	maker-chr9-exonerate_est2genome-gene-92.4	yes|down	1
OV80031997.g	maker-chr8-snap-gene-89.3	yes|down	1
OV100028327.g	maker-chr10-snap-gene-207.0	yes|down	1
OV50018111.g	maker-chr5-snap-gene-33.50	yes|down	1
OV90000681.g	maker-chr9-augustus-gene-141.0	yes|down	1
OV10024361.g	maker-chr1-augustus-gene-564.8	yes|down	1
OV140017068.g	maker-chr14-snap-gene-83.2	yes|down	1
OV30007935.g	maker-chr3-snap-gene-128.19	yes|up	1
